# Chromosomal translocation t(11;14) and *p53* deletion induced by the CRISPR/Cas9 system in normal B cell-derived iPS cells

**DOI:** 10.1038/s41598-021-84628-5

**Published:** 2021-03-04

**Authors:** Yusuke Azami, Naohiro Tsuyama, Yu Abe, Misaki Sugai-Takahashi, Ken-ichi Kudo, Akinobu Ota, Karnan Sivasundaram, Moe Muramatsu, Tomonari Shigemura, Megumi Sasatani, Yuko Hashimoto, Shigehira Saji, Kenji Kamiya, Ichiro Hanamura, Takayuki Ikezoe, Masafumi Onodera, Akira Sakai

**Affiliations:** 1 Department of Medical Oncology, Fukushima Medical University School of Medicine, Fukushima, 960-1295 Japan; 2 Department of Radiation Life Sciences, Fukushima Medical University School of Medicine, 1 Hikarigaoka, Fukushima, 960-1295 Japan; 3 Department of Hematology, Aichi Medical University School of Medicine, Nagakute, 480-1195 Japan; 4 Department of Diagnostic Pathology, Fukushima Medical University School of Medicine, Fukushima, 960-1295 Japan; 5 Department of Pediatrics, Shinshu University, Matsumoto, 390-8621 Japan; 6 Department of Experimental Oncology, RIRBM, Hiroshima University, Hiroshima, 734-8553 Japan; 7 Department of Hematology, Fukushima Medical University School of Medicine, Fukushima, 960-1295 Japan; 8 Department of Genetics, National Research Institute for Child Health, Development, Tokyo, 157-8535 Japan

**Keywords:** Cell biology, Stem cells

## Abstract

Multiple myeloma (MM) cells are derived from mature B cells based on immunoglobulin heavy chain (*IgH*) gene analysis. The onset of MM is often caused by a reciprocal chromosomal translocation (cTr) between chr 14 with *IgH* and chr 11 with *CCND1*. We propose that mature B cells gain potential to transform by reprograming, and then chromosomal aberrations cause the development of abnormal B cells as a myeloma-initiating cell during B cell redifferentiation. To study myeloma-initiating cells, we have already established normal B cell-derived induced pluripotent stem cells (BiPSCs). Here we established two BiPSCs with reciprocal cTr t(11;14) using the CRISPR/Cas9 system; the cleavage site were located in the *IgH* Eμ region of either the VDJ rearranged allele or non-rearranged allele of *IgH* and the 5′-upsteam region of the *CCND1* (two types of BiPSC13 with t(11;14) and MIB2-6 with t(11;14)). Furthermore, *p53* was deleted using the CRISPR/Cas9 system in BiPSC13 with t(11;14). These BiPSCs differentiated into hematopoietic progenitor cells (HPCs). However, unlike cord blood, those HPCs did not differentiated into B lymphocytes by co-culture with BM stromal cell. Therefore, further ingenuity is required to differentiate those BiPSCs-derived HPCs into B lymphocytes.

## Introduction

The cellular origin of multiple myeloma (MM) has not been identified. Based on the experiments of transplantation of bone marrow (BM) samples from MM patients into immunodeficient mice, so-called myeloma stem cells have been inferred to be present in CD19^−^/CD38^++^/CD138^+^ or CD138^−^ plasma cell populations^1,2^; however, the results may indicate the presence of plasma cell populations with cell proliferation ability rather than the cellular origin of myeloma cells. On the other hand, based on immunoglobulin heavy chain (*IgH*) gene analysis, myeloma cells are derived from post germinal centre B cells^3^ (Figure S1). Chromosomal aberrations such as trisomy and chromosomal translocation (cTr) play a critical role in early tumorigenesis of MM^4,5^. We established induced pluripotent stem (iPS) cells from normal B lymphocytes (BiPSCs: BiPSC13 and MIB2-6) to test the hypothesis that the abnormal cells of origin responsible for tumorigenesis of MM are reprogrammed mature B lymphocytes^6^, and these BiPSCs have the same VDJ rearrangement of *IgH* as the original B lymphocytes and differentiate into CD34^+^/CD38^−^ hematopoietic progenitor cells (HPCs) when co-cultured with stromal cells. Furthermore, these cells can induce the expression of activation-induced cytidine deaminase (AID) that causes mutations not only in *IgH* but also in other genes, and further causes double strand breaks in DNA.

Here we generated BiPSCs with reciprocal cTr t(11;14), which is reciprocal translocation between *IgH* and *CCND1* and the most frequent cTr in MM^4,5^, using the clustered regularly interspaced short palindromic repeats (CRISPR)/Cas9 system^7^. Furthermore, we generated BiPSC13 with t(11;14) with a deletion in exon 5 of *p53* because deletion of *p53* is involved in the progression of MM^4,5^ (Figure S2). Subsequently, we analyzed the features of cTr t(11;14) between the functional allele or the non-functional allele of *IgH* and *CCND1* and the ability to differentiate into blood cells.

## Results

### Construction of IgH/cyclin D1-specific CRISPR/Cas9

The *CCND1* cleavage site on chr 11 was upstream of *CCND1* where off-target effects are fewer and setting a protospacer adjacent motif (PAM) site is easy, based on a study showing no hot spots at the cleavage site in the analysis of MM patients^8^. The *IgH* cleavage site on chr 14 was targeted at a site with fewer off-target effects, between Eμ and Iμ in the class switch region of *IgH,* and where setting a PAM site is easy^9^. We then designed a CRISPR/Cas9 vector in which these were arranged in tandem to obtain artificial induction of t(11;14) by cutting *CCND1* upstream on chr 11 and between Eµ and Iµ regions of *IgH* on chr 14. We designed two efficient gRNA sequences to be expressed in one CRISPR/Cas9 vector^7^. The functioning of this system was confirmed by induction of cTr t(11;14) in 293 T cells^7^. We attempted to induce reciprocal cTr t(11;14) in two normal B lymphocyte-derived iPS cell lines (BiPSC13, MIB2-6) using the above method. In both BiPSC13 and MIB2-6, *IgH* has a complete VDJ rearrangement (VH3-FR1: V_3-9_D_4-23_J_2_ in BiPSC13, VH4-FR1: V_4-39_D_3-22_J_6_ in MIB2-6), no class switch recombination (CSR) of *IgH* (Supplemental information, Figure S3), and no somatic hypermutations (SHM) in the VDJ region compared to germline (Supplemental information, Figure S4A and S4C).

**Figure 1:**
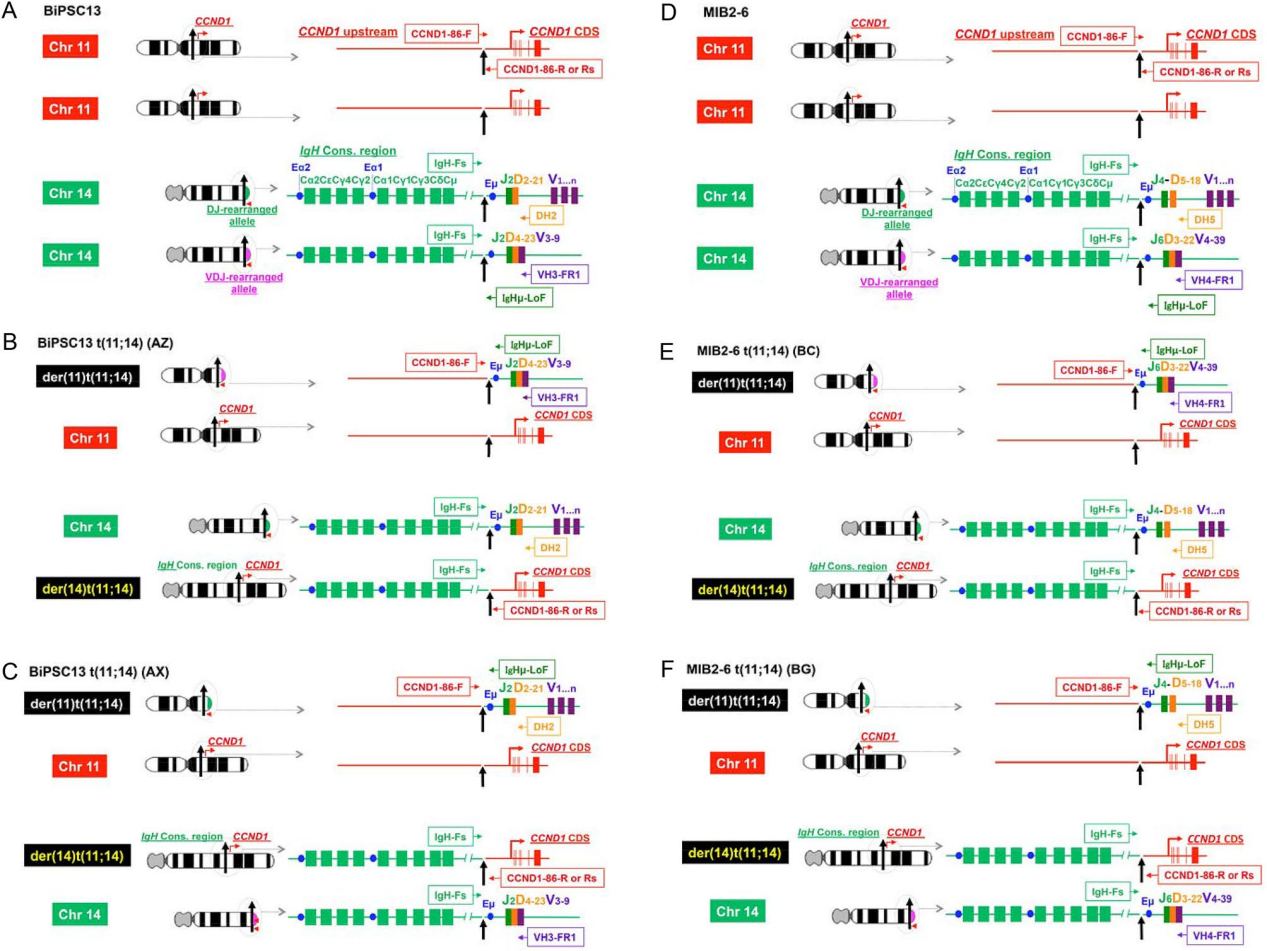

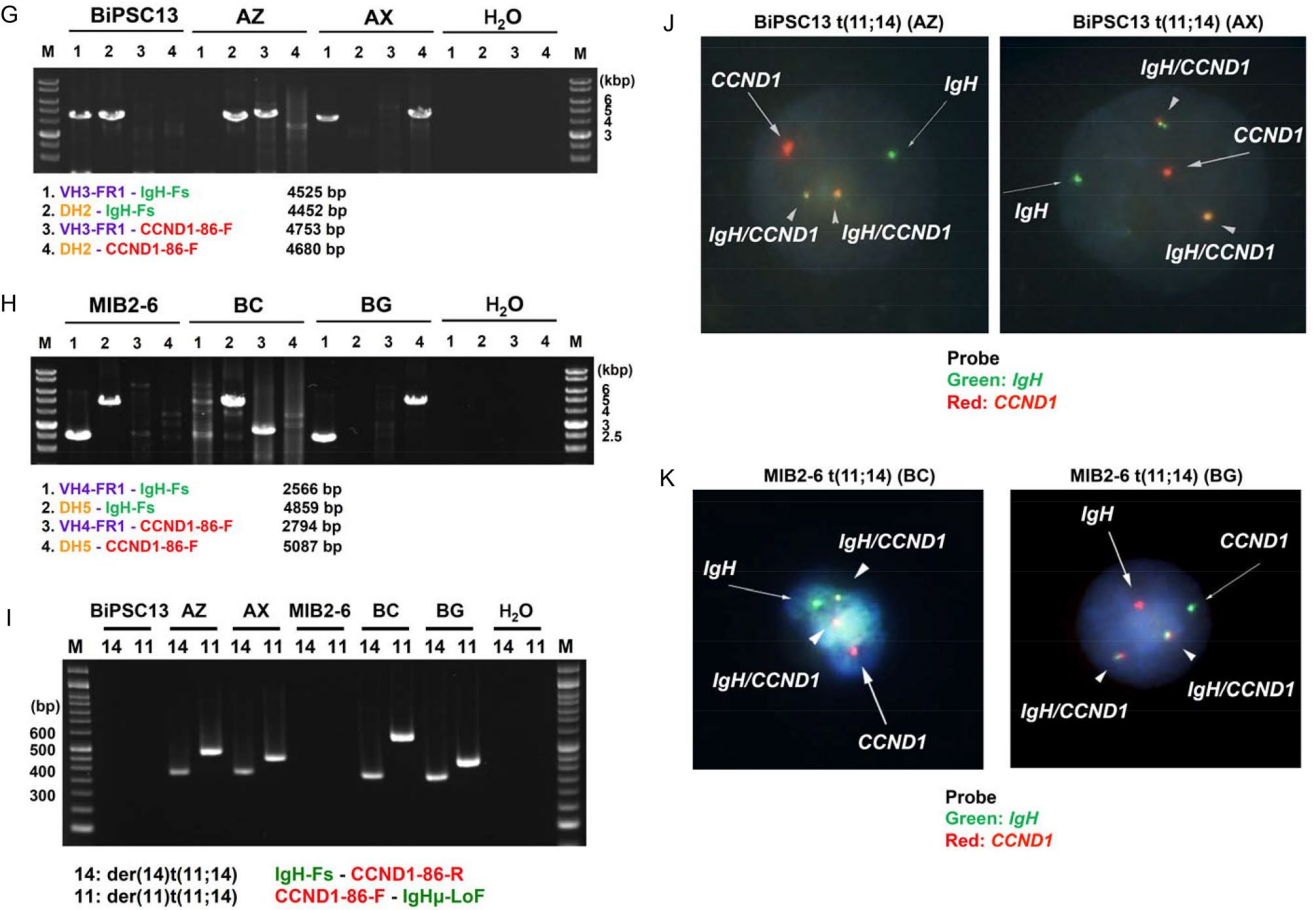

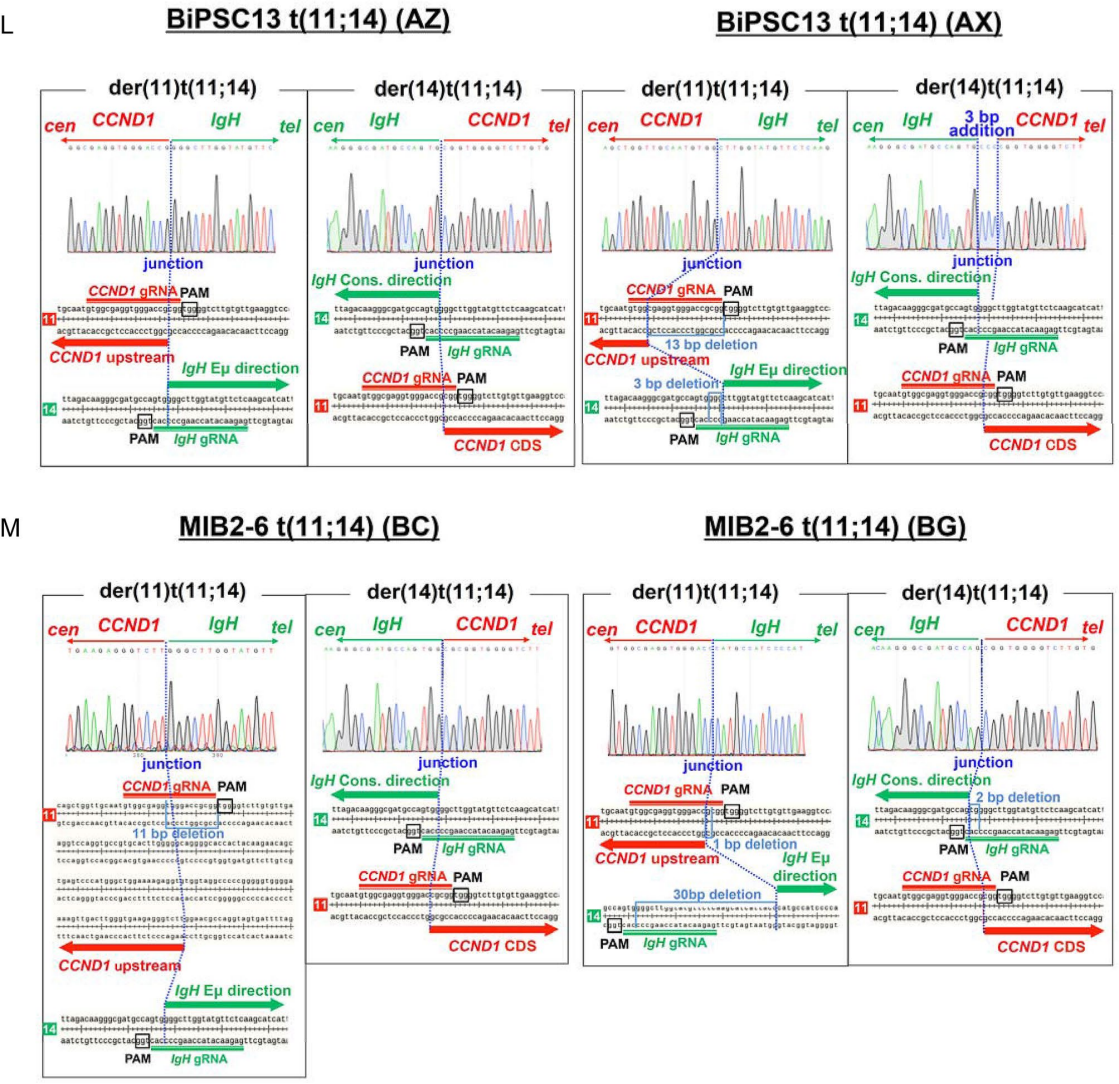
Features of the reciprocal chromosomal translocation (cTr) between *CCND1* on chr 11 and *IgH* on chr 14. **(A, D)** Two pairs of *IgH* on chr 14 and *CCND1* on chr 11 of BiPSC13 and MIB2-6 are shown. The cleavage site of CRISPR/Cas9 is indicated by (**↑**). The bent arrows and arrowheads indicate the direction of transcription of *CCND1* and *IgH*, respectively. CCND1-86F, CCND1-86R, IgH-Fs, DH2, DH5, VH3-FR1, VH4-FR1, IgHu-LoF represent PCR primers for the confirmation of cTr t(11;14). **(B)** Reciprocal cTr between the functional allele (V_3-9_D_4-23_J_2_) of *IgH* and *CCND1* in BiPSC13 (AZ). **(E)** Reciprocal cTr between the functional allele (V_4-39_D_3-22_J_6_) of *IgH* and *CCND1* in MIB2-6 (BC). **(C)** Reciprocal cTr between the non-functional allele (D_2-21_J_2_) of *IgH* and *CCND1* in BiPSC13 (AX). **(F)** Reciprocal cTr between the functional allele (D_5-18_J_4_) of *IgH* and *CCND1* in MIB2-6 (BG). **(G)(H)** Confirmation of reciprocal cTr between *IgH* and *CCND1* with PCR. PCR in BiPSC13 and two types of BiPSC13 with t(11;14) (AZ and AX) (G) and PCR in MIB2-6 and two types of MIB2-6 with t(11;14) (BC and BG) (H). Refer to (A) and (D) above for the location of each primer. The numbers indicate the length of the PCR product estimated from the database. **(I)** Confirmation of der(14)t(11;14) and der(11)t(t(11;14) in AZ, AX, BC, and BG with PCR. Refer to (A) and (D) above for the location of each primer. **(J,K)** Chromosomal t(11;14)-specific FISH of AZ and AX (J), and BC and BG (K). One chr 11 and chr 14 was reciprocally translocated. *CCND1* and *IgH* probes are labeled with red and green boxes, respectively. The arrowheads indicate the *IgH-CCND1* fusion signal. **(L,M)** Reciprocal cTr t(11;14) junction sequence of AZ and AX (L), and BC and BG (M). The dotted line indicates the cleavage site (usually three bases from the PAM region). Details are described in the results.

Two pairs of *IgH* on chr 14 and *CCND1* on chr 11 of BiPSC13 and MIB2-6 are shown in Fig. 1A,D. In BiPSC13, the upper and lower alleles of chr 14 show the non-functional allele that stopped at DJ rearrangement (D_2-21_J_2_) and the functional allele that completed VDJ rearrangement (V_3-9_D_4-23_J_2_) of *IgH*, respectively (Figure S4A and S4B). In MIB2-6, the upper and lower alleles of chr 14 show the non-functional allele in which DJ rearrangement was incomplete (D_5-18_J_4_), and the functional allele in which VDJ rearrangement was complte (V_4-39_D_3-22_J_6_) for *IgH*, respectively (Figure S4C and S4D). The DJ joining of the non-functional allele of MIB2-6 was incomplete because DH4, DH6, and DH7 were deleted between D_5-18_ and J_4_, but an intron of 1000 bases or more remained (Figure S4D).

Using the CRISPR/Cas9 system, we induced two kinds of reciprocal cTr t(11, 14), in which the translocation allele of *IgH* was different, in BiPSC13 and MIB2-6 (BiPSC13 t(11;14) and MIB2-6 t(11;14), respectively). AZ and AX were the different types of BiPSC13 t(11;14) carrying either the VDJ-rearranged allele or the DJ-rearranged allele of *IgH, respectively,* that reciprocally translocated with *CCND1* (Fig. 1B,C). BC and BG were the different types of MIB2-6 t(11;14) carrying either the VDJ-rearranged allele or the DJ-rearranged allele of *IgH, respectively,* that reciprocally translocated with *CCND1* (Fig. 1E,F).

The features of these cTrs were confirmed with PCR using translocation-specific primers (Fig. 1). The PCR product in lane 1 shows the presence of the VDJ-rearranged functional allele of *IgH*, and the PCR product in lane 2 shows the non-functional allele that stopped at DJ rearrangement of *IgH*. Therefore, the PCR product in lane 3 shows the translocation between the functional allele of *IgH* and *CCND1*, and the PCR product in lane 4 shows the translocation between the non-functional allele of *IgH* and *CCND1* (Fig. 1G,H). Among these cTrs, when the VDJ side of the *IgH* functional allele translocated with *CCND1* upstream, der(11)t(11;14) was formed. Simultaneously, the CDS side of *CCND1* translocated with the constant region of *IgH,* and der(14)t(11;14) was formed (AZ and BC) (Fig. 1B,E,G–I). On the other hand, when the DJ side of the non-functional allele of *IgH* translocated with *CCND1* upstream, der(11)t(11;14) was formed. Simultaneously, the CDS side of *CCND1* translocated with the constant region of *IgH,* and der(14)t(11;14) was formed (AX and BG) (Fig. 1C,F,G–I). Therefore, we established cell lines carrying reciprocal cTr t(11;14) between *CCND1* and either an allele in which VDJ rearrangement of *IgH* was completed or an allele in which VDJ rearrangement was not completed (stopped at DJ joining) in BiPSC13 t(11;14)(AZ and AX)and MIB2-6 t(11;14) (BC and BG), respectively. We also confirmed the reciprocal cTr t(11;14) using IGH-CCND1 FISH (Fig. 1J,K).

Subsequently, we analyzed the nucleotide sequences near the translocation sites of AZ and AX, and BC and BG (Fig. 1L,M). Thirteen-base deletion of the *CCND1* gRNA region and three-base deletion of the *IgH* gRNA region in der(11)t(11;14) of AX, and three-base addition at the translocation junction in der(14)t(11;14) of AX were confirmed (Fig. 1L). The cleavage site was 140 bases downstream from the PAM region of *CCND1* gRNA in der(11)t(11;14) of BC, and 11-base deletion in the *CCND1* gRNA region was confirmed (Fig. 1M). One-base deletion in the *CCND1* gRNA and 30-base deletion in the Eμ direction from the *IgH* gRNA in der(11)t(11;14) of BG, and 2-base deletion in the *IgH* gRNA region in der(14)t(11;14) of BG were confirmed (Fig. 1M).

Effects of CRISPR/Cas9 on alleles of *IgH* not used in the translocation with *CCND1* and on alleles of *CCND1* not used in the translocation with *IgH* were also analyzed (Supplemental information, Figure S5 and S6). Deletions from 11 to 333 bases were found around the cleavage sites of Cas9 in those BiPSCs.

### Knockout of TP53 with the CRISPR/Cas9 system

**Figure 2:**
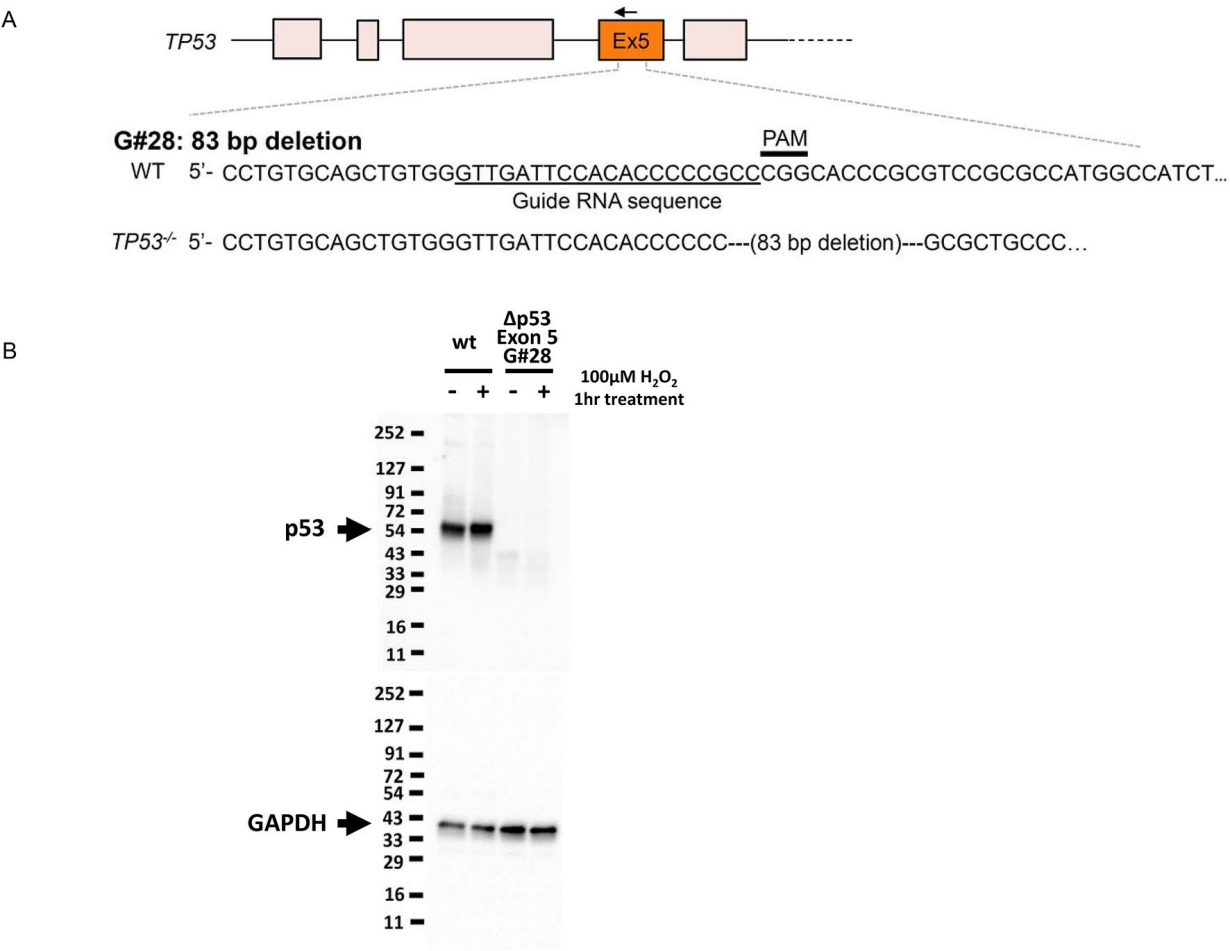
Knockout of *p53* with the CRISPR/Cas9 system. **(A)** Induction of 83 bp deletion in exon 5 of *p53* using the CRISPR/Cas9 system. **(B)** Confirmation of suppression of *p53* expression in G#28 with western blotting. Induction of apoptosis by H_2_O_2_ enhanced TP53 expression in wt (BiPSC13), but TP53 expression was not observed in G#28 regardless of H_2_O_2_ treatment.

Because deletion of chr 17p, including *p53* deletion, is involved in the progression of MM, genome editing was performed on *p53* in BiPSC13 t(11;14) (AX) using the CRISPR/Cas9 system. The expression of TP53 was knocked out by deleting 83 bp of exon 5 of *p53* (G#28: Fig. 2). Subsequently, we established G#28 and AX with AID expression regulated by the doxycycline-controlled (Tet-off) system (G#28-AID and AX-AID)^6^ (Supplemental information, Figure S7).

### Differentiation of BiPSCs into blood cells

**Figure 3:**
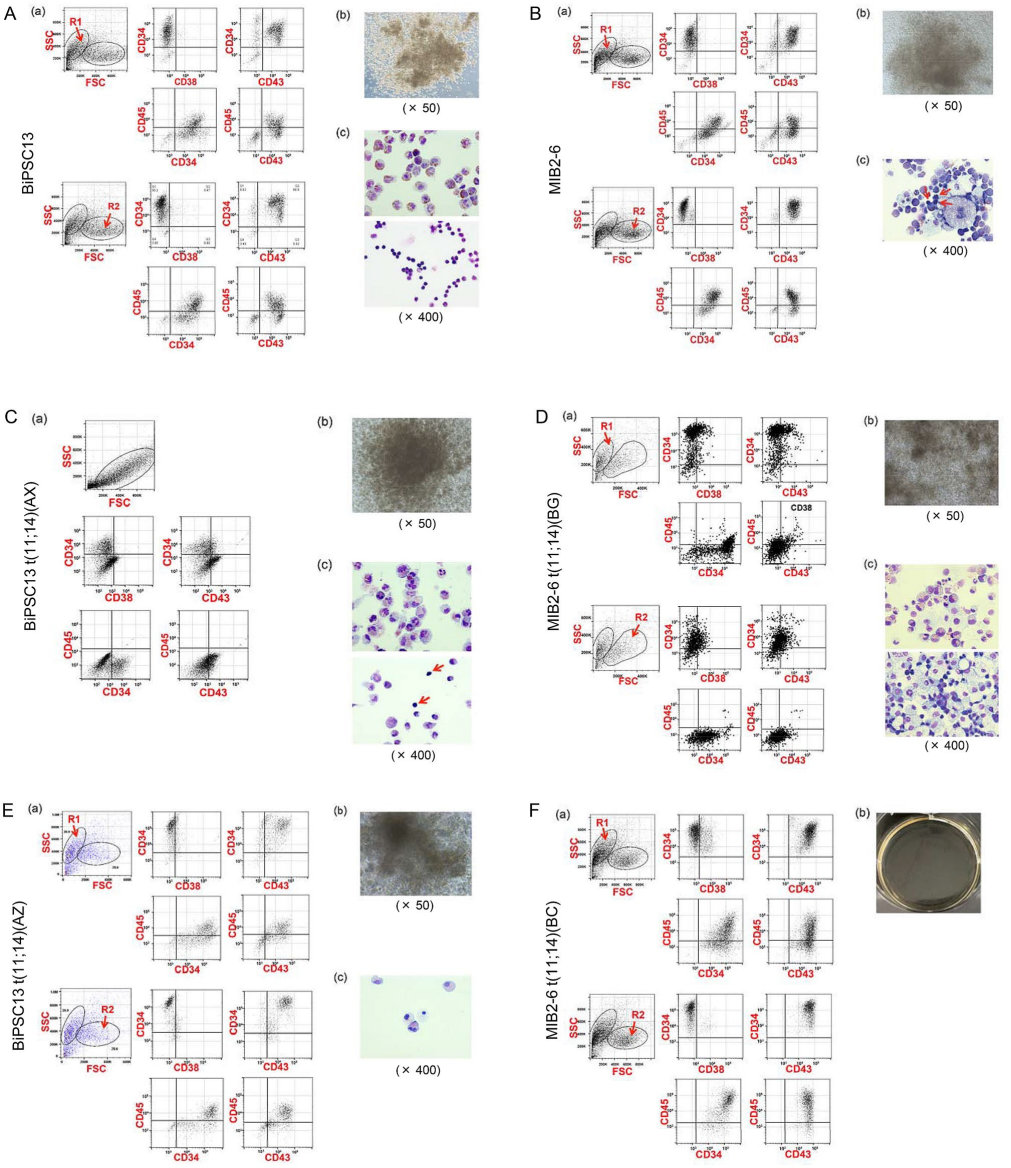

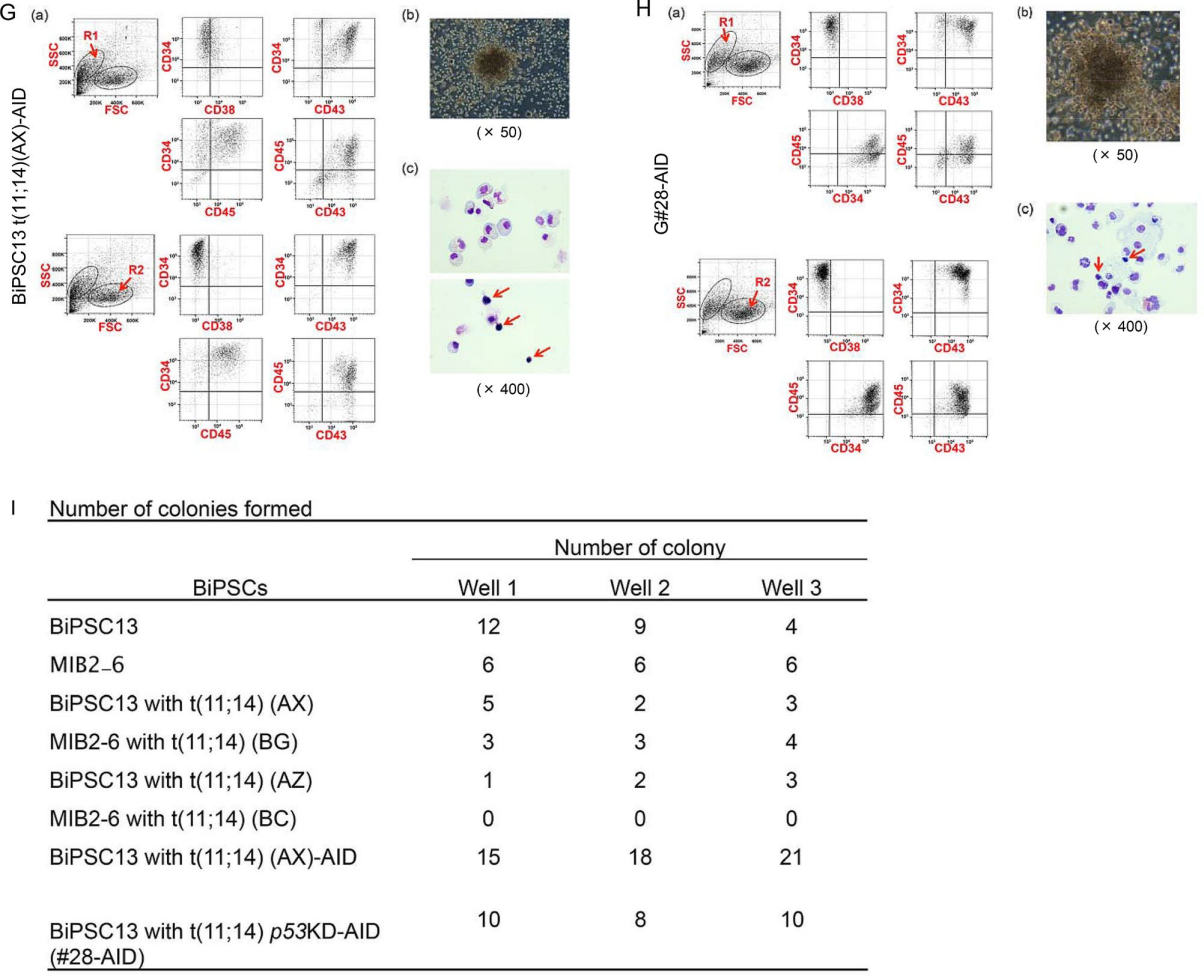
Induction of differentiation of parent BiPSCs and their genome-edited BiPSCs into blood cells. **(A)** BiPSC13, **(B)** MIB2-6, **(C)** AX, **(D)** BG), **(E)** AZ, **(F)** BC, **(G)** AX with AID, and **(H)** G#28 with AID. (a) Flow cytometric analysis of the cell phenotype after differentiation of BiPSCs into HPCs. Two cell populations were observed (R1 and R2). (b) Representative morphology of formed colonies (× 50). (c) Wright staining of cytospins picked up from a colony (× 400). The bottom picture of (A) and arrows indicate erythrocytes. The bottom picture of (D) shows a mix of macrophages and erythrocytes. **(I)** Number of colonies formed. The types of colonies formed were assessed in triplicate.

Initially, the induction of differentiation into HPC was performed by co-culturing BiPSC13, MIB2-6 with AGM-S3^10^ (Supplemenatal information, Figure S8). However, the differentiation efficiency was not stable, and AGM-S3 contamination was present even after purification using CD34 microbeads. We then used the STEMdiff Hematopoietic Kit to induce differentiation of BiPSCs into HPCs (Fig. 3). Based on a model of hematopoietic differentiation from human embryonic stem cells^11^, the expression of CD43, CD45, and CD38 was examined in CD34^+^ cells. Except for AX (Fig. 3C), two cell populations were observed (R1 and R2), and most CD34^+^ cells were CD43^+^/CD45^+^/CD38^+/−^ in both populations (Fig. 3). The number of colonies formed in the colony assay is shown in Fig. 3I. Most colonies were a mix of macrophages and granulocytes, and some were mixed with erythrocytes (Fig. 3A–D,G,H). BC differentiated into CD34^+^ cells, but colony formation was not observed (Fig. 3F). On the other hand, differentiation into blood cells was observed even in the case of *p53* deletion and induction of AID expression in the colony assay in AX (Fig. 3G,H). Therefore, B cell-derived iPS cells were able to differentiate into HPCs even in the presence of cTr t(11;14) or *p53* was deletion.

### Differentiation of BiPSCs-derived CD34^+^ cells and cord blood (CB) into B lymphocyte

**Figure 4:**
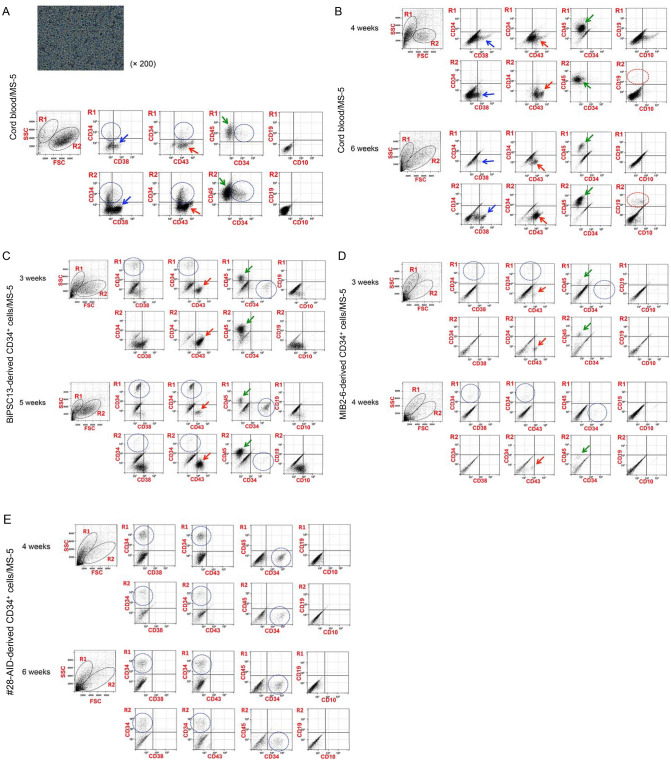
Induction of differentiation into B lymphocytes by co-culture with MS-5. **(A)** Ten days after starting co-culture of cord blood (CB) and MS-5. The photo reveals a growth of CB on MS-5. Phenotype analysis of non-adherent floating cells harvested after gentle agitation. CD34^+^ cells are circled with blue dotted lines. Blue arrows, red arrows, and green arrows indicate CD38^+^ cells, CD43^+^ cells, and CD45^+^ cells, respectively. **(B)** Phenotype analysis of mixed floating and adherent cells 4 and 6 weeks after initiating the co-culture of CB and MS-5. The cell population indicated by each arrow is the same as in (A). CD19^+^ cells are circled with red dotted lines. **(C)** Phenotype analysis of mixed floating and adherent cells 3 and 5 weeks after initiating the co-culture of BiPSC13-derived CD34^+^ cells and MS-5. CD34^+^ cells are circled with blue dotted lines. Red arrows and green arrows indicate CD43^+^ cells and CD45^+^ cells, respectively. **(D)** Phenotype analysis of mixed floating and adherent cells 3 and 4 weeks after initiating the co-culture of MIB2-6-derived CD34^+^ cells and MS-5. The cell populations indicated by blue dotted lines and arrows are the same as in (C). **(E)** Phenotype analysis of mixed floating and adherent cells 4 and 6 weeks after initiating the co-culture of G#28-AID-derived CD34^+^ cells and MS-5. CD34^+^ cells are circled with blue dotted lines.

We investigated whether HPCs differentiated from iPS cells derived from normal B lymphocytes can differentiate into B lymphocyte again by co-culture with MS-5, with reference to a study by MacLean^12^. First, we confirmed that CB would differentiate into B lymphocytes by co-culture with MS-5, with reference to studies by Nishihara^13^ and Hirose^14^. CB proliferated rapidly after initiating the co-culture, and phenotype analysis at day 10 revealed that CD34^+^ cells were mainly present in the R2 region (Fig. 4A). The main cell population in both the R1 and R2 regions was CD34^−^/CD38^+/−^/CD43^+^/CD45^+^, followed by CD34^+^/CD38^−^/CD43^+^/CD45^+^ (Fig. 4A). Four weeks after initiating the co-culture, the CD34^+^ cells had disappeared and the main cell populations were CD34^−^/CD38^+/−^/CD43^+^/CD45^+^, and CD19^+^/CD10^−^ cells were observed in R2 region (Fig. 4B). The number of CD19^+^/CD10^−^ cells further increased 6 weeks after initiation of the co-culture (Fig. 4B).

Next, we investigated whether two types of BiPSCs (BiPSC13 and MIB2-6) would differentiate into B lymphocytes by co-culture with MS-5. The cells were differentiated into CD34^+^ cells using a STEMdiff Hematopoietic kit, and purified CD34^+^ cells were subsequently cultured on MS-5. Phenotype analysis was performed 3 weeks after initiation of the co-culture, with reference to a previous study^12^. Based on studies reporting the appearance of CD19^+^ cells from the fourth week of co-culture of CB with MS-5^13,14^, we performed phenotype analyses 4 or 5 weeks after initiation of the co-culture. Analysis of BiPSC13-derived cells showed a non-specific cell population, even after staining with an isotype control using FITC or without staining (Figure S9). Therefore, the cell population observed by the double staining of CD34/CD38 and CD19/CD10 was likely to be due to autofluorescence. In the co-culture of BiPSC13 or MIB2-6-derived CD34^+^ cells with MS-5, the CD34^+^ cells remained mainly in the R1 region and their phenotype were CD34^+^/CD38^−^/CD43^−^/CD45^−^ (Fig. 4C,D). A population of CD34^−^/CD38^−^/CD43^+^/CD45^+^ cells was also observed (Fig. 4C,D). No CD19^+^ cells were observed. In the co-culture of G#28-AID-derived CD34^+^ cells with MS-5, CD34^+^ cells were still found in both the R1 and R2 regions and their phenotype was CD34^+^/CD38^−^/CD43^−^/CD45^−^ (Fig. 4E).

Therefore, the critical difference between these three types of BiPSCs-derived HPCs and CB was that the former CD34^+^ cells remained and did not differentiate into CD38^+^ cells even after 4 weeks of co-culture with MS-5.

## Discussion

We hypothesized that the abnormal cells from which myeloma cells originate may be mature B lymphocytes with chromosomal or genetic changes in the reprogrammed state that enable them to acquire the potential to become tumor cells during the process of redifferentiation into B lymphocytes. Here we tried to generate cells to test this hypothesis. First, we established iPS cells from normal B lymphocytes (BiPSCs) that can induce AID expression^6^. We then generated BiPSCs carrying the most frequent reciprocal cTr t(11;14) in MM (BiPSC13 t(11;14) and MIB2-6 t(11;14)), and also generated BiPSC13 t(11;14) with *p53* deletion, which is involved in the progression of MM. This means “Strike (chromosomal or genetic changes) while the iron (B lymphocytes) is hot”.

Studies have reported the possibility of generating reprogrammed tumor cells or precancerous lesions by forcibly expressing the Yamanaka factors in colonic epithelium or pancreatic acinar cells that already have gene mutations^15,16^. Interestingly, in vivo reprograming is possible in the presence of inflammation and senescence instead of Yamanaka factors, and depolarization of the original cell is considered to be critical^17^. We believe that normal B lymphocytes could be transformed into iPS-like cells in the environment of BM or lymph nodes in which Yamanaka factors are induced due to chronic inflammation. The original cells of BiPSCs were normal cells, unlike the cells mentioned above^15,16^, and genomic changes were induced after transformation into iPS cells.

The onset of MM is often caused by a reciprocal cTr between chr 14 with *IgH* and chr 11 with *CCND1* or chr 4 with *FGFR3* and *MMSET*^4,5^. In follicular lymphoma (FL) and mantle cell lymphoma (MCL), immature B lymphocytes in BM are considered the origin of tumor cells; the reciprocal cTr between *IgH* and oncogenes such as *BCL-2* on chr 18 or *CCND1* on chr 11 during VDJ rearrangement is responsible for tumorigenesis of B lymphocytes^18^. On the other hand, in MM, mature B lymphocytes, so-called plasma cells, or antibody-producing cells, are considered tumor cells because *IgH* reciprocally translocate with other genes during CSR^19,20^, and SHM is recognized in the VDJ region of *IgH*^21–23^. Considering the presence of M-protein, a functional allele of *IgH* is present in MM. Therefore, cTr occurs between another non-functional allele of *IgH* in which VDJ rearrangement has not been completed, and other chromosomes in MM^3^. The features of cTr in AX and BG exactly mimic that of MM.

When VDJ rearrangement of one allele of *IgH* is complete, VDJ rearrangement of another allele of *IgH* is not complete as a non-functional allele due to allelic exclusion^24,25^. In FL and MCL, cTr occurs during VDJ rearrangement in immature B lymphocytes as mentioned above. However, considering that B-cell receptors are expressed on the cell surface, the allele that initiates VDJ rearrangement earlier would be a non-functional allele due to cTr, and in turn, another allele that should have been non-functional, would undergo VDJ rearrangement to create a functional allele that expresses B-cell receptors. Although cTr during VDJ rearrangement in MM was also reported^26^, many cTrs occur during CSR^4,5^.

Even in immature B lymphocytes, cTr t(14;18) or cTr t(11;14) alone is believed to not be able to transform these B lymphocytes, as suggested by studies revealing these chromosomal aberrations in normal individuals^27–29^. Similarly, mature B lymphocytes would be unlikely to transform into tumor cells by cTr. Myeloma cells could be derived from reprogramed mature B lymphocytes with the following features: the CD19 antigen on the cell surface and its transcription factor, Pax5, are deleted in myeloma cells unlike in other B cell lymphomas^30^. Furthermore, myeloma cells expressing CD33^31^, which is a cell surface marker of granulocytes, or producing amylase^32,33^ or ammonia^34^ have been reported.

Myeloma cells are derived from mature B lymphocytes because of SHM in the VDJ region of *IgH* (Figure S10). Because neither SHM in the VDJ region nor CSR of *IgH* occurred in BiPSC13 or MIB2-6, those BiPSCs are considered to be derived from pre-germinal center (GC) B lymphocytes rather than mature B lymphocytes. However, they can induce AID expression with the tet-off system^6^, and we confirmed that those BiPSCs and BiPSCs with t(11;14) can differentiate into HPCs in this study. If they further redifferentiate into B lymphocytes, SHM in the VDJ region and CSR of the functional allele of *IgH* could be induced during the redifferentiation process by activation of endogenous AID. Furthermore, activation of enhancers involved in *IgH* expression during the process of redifferentiation into B lymphocytes would lead to overexpression of *CCND1* induced by cTr t(11;14), which is observed in MM with t(11;14). Expression of AID also would be expected to induce SHMs on various genes other than *IgH*, which would lead to the branching pathway theory based on a Darwinian selection perspective^35^, a model for the progression of MM. In addition, if *IgH* expression is impossible due to a deletion in the constant region instead of CSR in the functional allele, this would be considered a mechanism of development of Bence-Jones-type MM; only the light chain of the M-protein is detected in the urine (and serum), because myeloma cells in these cases do not produce the IgH chain.

Unfortunately, no CD19^+^ cells were observed in co-culture of BiPSCs-derived CD34^+^ cells with MS-5. The difference between these BiPSCs-derived HPCs and CB was that the former CD34^+^ cells remained and CD34^−^/CD43^+^/CD45^+^ cells did not express CD38 even after 4 weeks of co-culture with MS-5. Especially, compared with the parent cell (BiPSC13), G#28-AID did not differentiate into CD34^−^/CD43^+^/CD45^+^ cells with remaining of CD34^+^ cells even 6 weeks after the initiation of co-culture with MS-5. A deletion of *p53* might be involved in this feature. Given that CD38 is involved in lymphocyte activation^36,37^, the ability to express CD38 in differentiation from HPC could be important for differentiation into B lymphocytes. Considering the results of the colony assay, a differentiation potential into blood cells of BiPSCs-derived HPCs can be expected, so other factors could be required for co-culture with MS5 in order to differentiate them into B lymphocytes in vitro. Moreover, important questions are whether these BiPSCs with cTr or *p53* deletion are capable of forming MM or B lymphoid tumor cells, and whether AID induction induces additional genetic aberrations. We are planning to transplant these BiPSC-derived HPCs into BM of immunodeficienct mice.

## Materials and methods

### Materials

Plasmids used in this study, lentiCRISPRv2 (#98,290) were obtained from Addgene (www.addgene.org). lentiCRISPRv2 was a gift from Brett Stringer. Synthetic oligonucleotides and PCR primers were purchased from Eurofin Genomics (Tokyo, Japan). DNAiso was purchased from Takara bio (Kyoto, Japan). Thunderbird SYBR qPCR Mix and KOD-FX Neo were obtained from Toyobo (Tokyo, Japan). PEImax 40,000 was obtained from Polyscience Inc. (Warrington, PA, USA).

### Normal B cell-derived iPS cell (BiPSCs: BiPSC13, MIB2-6) culture

Established BiPSCs were maintained in a six-well plate coated with iMatrix-511 (Nippi, Tokyo, Japan) in BiPSC culture medium, StemFit AK02N (REPROCELL, Yokohama, Japan). The BiPSC culture medium was changed every day until the start of differentiation experiment using the STEMdiff Hematopoietic Kit (STEMCELL Technologies, Vancouver, Canada).

### Induction of translocation

cTr was induced by infection of BiPSCs with *IgH-CCND1* lentiCRISPRv2 lentivirus which targets the human *IgH* Eµ region and 13 kb upstream of the *CCND1* coding sequence (CDS)^7^. Briefly, CRISPRscan (http://www.crisprscan.org/)^38^ estimated the gRNA candidate, 5′-GGAGAACATACCAAGCCCCAC-3′ for *IgH* (105,861,064 to 105,861,045 of NC_000014.9, chromosome (chr) 14, GRCh38.p12 Primary Assembly) and 5′-GGTGGCGAGGTGGGACCGCGG-3′ for *CCND1* (69,627,757 to 69,627,776 of NC_000011, chr 11, GRCh38.p12 Primary Assembly), which were recombined into the lentiCRISPRv2 BsmBI site. The *CCND1* gRNA expression unit of *CCND1*-lentiCRISPRv2 was cloned into *IgH*-lentiCRISPRv2 to form the *IgH*-*CCND1* lentiCRISPRv2 dual site targeting vector. The vector was packaged into lentivirus, and BiPSCs cultures were infected with the packaged virus in the presence of polybrene (4 µg/mL) for 1 day. Puromycin selection (0.25 µg/mL) started after 2 days. Ten days later, drug-resistant colonies were picked and mechanically divided into two portions using pipet tips; one half was cultured, and DNA was isolated from the other half using DNAiso kit. Confirmation of cTr was performed by PCR using the *IgH/CCND1* translocation-specific primer pair (IgH-Fs for the *IgH* side and CCND1-86-Rs for the *CCND1* side) (Fig. 1, Table S1) with Thunderbird SYBR qPCR Mix and a Light Cycler Nano (Loche Diagnostics, Basel, Switzerland). PCR conditions were 94 °C 120 s, 50 cycles of (94 °C 10 s, 62 °C 10 s, 72 °C 20 s), and 72 °C to 94 °C at 0.1 °C/sec for melting temperature measurement. The presence of Tr-positive clones was assessed based on the melting temperature of PCR-amplified DNA compared with positive control DNA, which was the product amplified from the *IgH* and *CCND1* genomic region with PCR and recombined into a translocation-mimic PCR template (5′-*IgH-CCND1*-3′)^7^.

### Detection of VDJ and DJ rearrangements in BiPSCs

Confirmation of the VDJ recombination profile was performed as described^39^. Briefly, VDJ regions from BiPSC genomic DNA were amplified with PCR using primer pairs of VHn-FR1 (n = 1–6) and JH consensus for the functional *IgH* allele, and DH regions were amplified using primer pairs of DHn (n = 1–7) and JH consensus for the non-functional *IgH* allele. Primers are listed in Table S1. PCR conditions are 95 °C 120 s, 45 cycles of (95 °C 30 s, 62 °C 15 s, 72 °C 40 s) using TB Green Fast qPCR Mix and LightCycler Nano. PCR products were electrophoresed in an agarose gel, and bands of the corresponding size were purified using FastGene Gel/PCR Extraction Kit (NIPPON Genetics) and sequenced with the same primers for PCR (FASMAC Co. Ltd.). Sequence data were analyzed using the IgBLAST database (https://www.ncbi.nlm.nih.gov/igblast/) to identify the subtype of every subdomain. An additional primer, DH5-18-F was used for DNA sequence of the non-functional *IgH* allele of MIB2-6.

### Identification of reciprocal chromosomal translocation t(11;14)

VH3-FR1 primer for BiPSC13 or VH4-FR1 primer for MIB2-6 was used in combination with the CCND1-86-F primer to detect reciprocal cTr, which formed der(11)t(11;14), between the functional *IgH* allele and CCND1. DH2 primer for BiPSC13 or DH5 primer for MIB2-6 was used with the CCND1-86-F to detect reciprocal cTr, which formed der(11)t(11;14) between the non-functional *IgH* allele and CCND1. The combination of IgH-Fs and CCND1-86-R or CCND1-86-Rs was used to detect another reciprocal cTr forming der(14)t(11;14). The presence of the untranslocated allele of *IgH* was confirmed with PCR in the combinations of DH2 or VH3-FR1 and IgH-Fs for BiPSC13 and DH5 or VH4-FR1 and IgH-Fs for MIB2-6, respectively. The presence of the untranslocated allele of *CCND1* was confirmed with PCR in the combination of CCND1-86-F and CCND1-86-R for both BiPSCs (Fig. 1A–F). Long PCR was performe**d** using KOD FX Neo DNA polymerase and LifeTouch (BIOER Tech., Hangzhou, P. R. China). PCR conditions were 94 °C 120 s, 35 cycles of (94 °C 10 s, 64 °C 10 s, 68 °C 3 min). PCR products were electrophoresed in agarose gels, and gel images were obtained in a ChemDoc XRS + Imaging System and Image Lab 4.1 software (BIO-RAD, Hercules, CA, USA). Exposure time to obtain images was automatically adjusted to detect faint bands by the software. For DNA sequencing, major bands were purified using FastGene Gel/PCR Extraction Kit (Nippon Genetics, Tokyo, Japan). DNA sequences were analyzed by FASMAC (Atsugi, Japan) using primers for PCR positioned at translocation junction sites. Obtained sequences were aligned to *IgH* or *CCND1* genome sequences to confirm sequence identity and alterations using ApE (http://jorgensen.biology.utah.edu/wayned/ape/) and NCBI Blast (https://blast.ncbi.nlm.nih.gov/). The existence of the non-translocated allele of *IgH* was also confirmed with PCR in the combination of VH3-FR1 primer for BiPSC13 or VH4-FR1 primer for MIB2-6 with IgH-Fs in the same condition of long PCR for the detection of cTr described above. All primers are listed in Table S1.

### TP53 knockout using the CRISPR/Cas9 system

The CRISPR/Cas9 system was used to disrupt expression of *TP53*^40^. The pSpCas9(BB)-2A-GFP (PX458) vector was a gift from Feng Zhang (Addgene plasmids # 48,138). In brief, a single guide RNA (sgRNA) sequence was selected using E-CRISP (http://www.e-crisp.org/E-CRISP/index.html). The sgRNA sequence for *TP53* Exon 5 was 5′-GTTGATTCCACACCCCCGCC, which corresponds to the sequence on the 3′ side of the initiation codon of Δ133p53. The plasmid expressing hCas9 and *TP53* sgRNA was prepared by ligating oligonucleotides into the BbsI site of PX458 (TP53ex5/PX458). To generate a *TP53* knockout clone, 1 μg TP53ex5/PX458 plasmid was nucleofected into BiPSC13 with t(11;14) (AX) (1 × 10^6^ cells) using a 4D-Nucleofector instrument (Lonza Japan, Tokyo, Japan). After 3 days, cells expressing GFP were sorted using FACSAria (BD Bioscience, Franklin Lakes, NJ, USA), and single-cell cloning was performed. A single clone was selected, expanded, and used for biological assays. For sequence analysis of *TP53* exon 5, the following primer set was used to amplify genomic DNA: 5′-TTGCAGGAGGTGCTTACACA and 5′-GAATTCTGAAGGTCTCGTCGT.

### Culture of BiPSCs in stem cell differentiation medium for differentiation into HPCs

Differentiation of BiPSCs into HPC was performed using STEMdiff Hematopoietic Kit (STEMCELL Technologies, Vancouver, Canada). BiPSCs were cultured on Matrigel hESC-Qualified Matrix (CORNING, Corning, NY, USA), incubated with Gentle Cell Dissociation Reagent (STEMCELL Technologies) at room temperature for 4–6 min, removed using a scraper, and then, 50 μm-sized pieces were transferred to a matrigel-coated 12-well plate in Complete StemFit AK02N (100 pieces/well). Differentiation experiments were performed according to the instructions of the kit. BiPSCs with AID were cultured in the presence of 10 ng/mL doxycycline (Takara Bio, Kusatsu, Japan) to prevent AID expression. Concurrently, CD34^+^ cells were purified using MACS CD34 MicroBeads (Miltenyi Biotec Inc., Auburn, CA, USA) according to the manufacturer’s instructions, and the collected cells were used for phenotype analysis and a colony-forming assay.

### Phenotype analysis

Purified CD34^+^ cells were evaluated by two-color and three-color flow cytometry after staining in phosphate-buffered saline without calcium chloride or magnesium chloride with the following monoclonal antibodies: anti-CD19-PE, anti-CD34-PE (BioLegend, San Diego, CA, USA), anti-CD38-FITC, anti-CD43-FITC, anti-CD10-FITC (BioLegend), and anti-CD45-PE/Cy5 (BioLegend). Immunofluorescence of the labeled cell membrane was evaluated using a flow cytometer (S3e Cell Sorter; BIO-RAD). Furthermore, phenotype analysis was performed after co-culture of CD34^+^ cells with MS-5^41^.

### Colony-forming assay

Purified CD34^+^ cells were then used in colony-forming assays using Methocult H4435 (STEMCELL Technologies) in triplicate. The types of colonies formed were assessed around day 14.

### cTr t(11;14)-specific fluorescence in situ hybridization (FISH)

cTr t(11;14)-specific FISH was performed by LSI Medience (LSI Medience Corporation, Tokyo, Japan).

### Western blot analysis

The method was described in our previous study^6^. Briefly, cells were lysed in lysis buffer (0.5% NP-40, 1% TritonX-100, 150 mM NaCl, and 1 mM EDTA in 20 mM Tris, pH 7.5) with a protease inhibitor cocktail (Nacalai Tesque, Kyoto, Japan) and PhosSTOP phosphatase inhibitor cocktail (Roche Diagnosis). After ten min on ice, the cell lysates were centrifuged at 14,000 rpm for 10 min at 4 °C and the supernatants were recovered. Protein content of every sample was measured by Bradford method, and protein of 70 µg aliquots were separated in a Mini-PROTEAN TGX precast Gel (BIO-RAD) and transferred to a nitrocellulose membrane (BIO-RAD). The membrane was incubated with the indicated antibodies and horseradish peroxidase (HRP)-labeled secondary antibodies, and the signal was visualized with enhanced Chemi-Lumi One Super (Nacalai Tesque) and detected by the ChemDoc XRS + (BIO-RAD). The primary antibodies used were anti-AICDA (Abcam Japan, Tokyo, Japan), p53 (DO-1) (Santa Cruz Biotech, Dallas, TX, USA), and anti-GAPDH (Abcam) and the second antibody was goat anti-mouse IgG-HRP or goat anti-rabbit IgG-HRP (Santa Cruz Biotech).

### Co-culture of purified CD34^+^ cells or cord blood (CB) with MS-5 for differentiation into B lymphocyte

The murine BM stromal cell line MS-5^41^ (a kind gift from Dr. Katsuhiko Itoh, Kyoto University), was maintained in α-MEM (Nacalai Tesque) supplemented with 20% horse serum (HS) (JRH (SAFC) Biosciences, Inc. Lenexa, KS, USA) and 1% penicillin/streptomycin (PS) (Nacalai Tesque). The cells were cultured to approximately 80% confluency in a FALCON tissue culture flask 50 mL (CORNING) for co-culture with purified CD34^+^ cells differentiated from BiPSC13, MIB2-6 and G#28-AID, or in a six-well tissue plate (Becton Dickinson, Franklin Lakes, NJ, USA) for co-culture with human CD34^+^ progenitor cells from cord blood (CB) (hCD34^+^-CB, single donor; Takara Bio). CD34^+^ cells (1 × 10^4^ cells/mL) derived from BiPSC13, MIB2-6 and G#28-AID, and 0.5 × 10^4^ cells/mL of CD34^+^ cells from CB, were plated onto the MS-5 feeder cells in α-MEM supplemented with 10% FBS, 1% GlutaMAX (Thermo Fisher Scientific KK, Tokyo, Japan), 1-thioglycerol (4 × 10^–4^ M, Sigma-Aldrich Japan, Tokyo, Japan), SCF (50 ng/mL) (BioLegend), G-CSF (25 ng/mL) (BioLegend), Flt3-L (50 ng/mL) (BioLegend), and IL-7 (20 ng/mL) (BioLegend). All cultures were performed in a humidified incubator containing 5% CO2 in air at 37 °C. Every 7 days, the cells were fed by removing half of the medium and replacing it with fresh medium containing fresh cytokines. Samples of CB co-cultured with MS-5 were collected after 10 days, 4 weeks and 6 weeks of co-culture, and the other samples were collected after 3–5 weeks of co-culture. For phenotype analysis, co-culture supernatant was collected, the MS-5 layer was incubated with collagenase IV (1 mg/mL) for 20 min at 37 °C, then the supernatant and MS-5 were combined and subjected to phenotype analysis.

## Supplementary Information

Supplementary Information
