# Supplementary material for: Chromosomal translocation t(11;14) and p53 deletion induced by the CRISPR/Cas9 system in normal B cell-derived iPS cells
Source: Sci Rep. 2021 Mar 4;11:5216. doi: 10.1038/s41598-021-84628-5 (PMC7933289; doi:10.1038/s41598-021-84628-5)
Supplement: Supplementary file 1 — Supplementary Information [file 41598_2021_84628_MOESM1_ESM.pdf]

Supplemental information

Chromosomal translocation t(11;14) and *p53* deletion induced by the CRISPR/Cas9 system in normal B cell-derived iPS cells

Running title: Genome editing in normal B cell-derived iPS cells

Yusuke Azami[1], Naohiro Tsuyama[2], Yu Abe[2], Misaki Sugai[2], Kenichi Kudo[2], Akinobu Ota[3], Karnan Sivasundaram[3], Moe Muramatsu[4], Tomonari Shigemura[5], Megumi Sasatani[6], Yuko Hashimoto[4], Shigehira Saji[1], Kenji Kamiya[6], Ichiro Hanamura[3], Takayuki Ikezoe[7], Masafumi Onodera[8], Akira Sakai[2]

[1]Dept. of Medical Oncology, Fukushima Med. Univ. School of Medicine, Fukushima, Japan
[2]Dept. of Radiation Life Sciences, Fukushima Med. Univ. School of Medicine, Fukushima, Japan
[3]Dept. of Hematology, Aichi Med. Univ. School of Medicine, Nagakute, Japan
[4]Dept. of Diagnostic Pathology, Fukushima Med. Univ. School of Medicine, Fukushima, Japan
[5]Dept. of Pediatrics, Shinshu Univ., Matsumoto, Japan
[6]Dept. of Experimental Oncology, RIRBM, Hiroshima Univ., Hiroshima, Japan
[7]Dept. of Hematology, Fukushima Med. Univ. School of Medicine, Fukushima, Japan
[8]Dept. of Genetics, National Research Institute for Child Health, Development, Tokyo, Japan

Correspondence:
Akira Sakai, M.D., Ph.D.
Dept. of Radiation Life Sciences
Fukushima Medical University School of Medicine
1 Hikarigaoka, Fukushima, 960-1295 Japan
Tel: +81-24-547-1420
Fax: +81-24-547-1940
E-mail: sakira@fmu.ac.jp

Materials and Methods

Copy number analysis of the *IgH* constant region (Iμ, Cμ, Cδ, Cγ3, and Cγ1)

DNA (50 ng) of BiPSC13 and MIB2-6 was PCR-amplified using TB Green Fast qPCR Mix (TOYOBO) and LightCycler Nano (Roche Diagnostics) with combinations of primers (0.5 μM) listed in Table S1 in the following conditions: 95°C 2 min, 55 cycles of (95°C 5 sec, 68°C 30sec), 68°C 60 sec, 64-95°C 0.1°C /sec. Ratio of 2-ΔΔCq values were calculated from the values obtained by LightCycler Nano Software[1].

PCR condition for the detection of VDJ and DJ rearrangements of *IgH* in BiPSC13 and MIB2-6

Genome DNA of BiPSC13 and MIB2-6 were amplified with PCR using primers listed in Table S1. PCR conditions are 95°C 120 sec, 45 cycles of (95°C 30 sec, 62°C 15 sec, 72°C 40 sec) using TB Green Fast qPCR Mix and LightCycler Nano. *IgH* V region primers, *IgH* D region primers, and *IgH* J consensus primers were from the previous study by van Dongen et al[2].

Nucleotide sequences of both alleles of *IgH* of BiPSC13 and MIB2-6

PCR products of VDJ and DJ rearrangements were electrophoresed in an agarose gel and purified. DNA sequences were analyzed by FASMAC (Kanagawa, Japan) using primers listed in Table S1.

PCR condition for the identification of reciprocal chromosomal translocation (Tr) t(11;14)

(1) PCR to identify der(11)t(11;14) or der(14)t(11;14) and the presence of the untranlocated *IgH* allele

Genome DNA of BiPSC13 and MIB2-6 with translocations were amplified with PCR using primers listed in Table S1. PCR conditions are 95°C 120 sec, 35 cycles of (95°C 1 sec, 64°C 10 sec, 72°C 180 sec) using KOD-Fx DNA polymerase and LifeTouch.

(2) PCR for the sequencing of der(11)t(11;14) or der(14)t(11;14) and the presence of the untranslocated *IgH* allele

Genome DNA of BiPSC13 and MIB2-6 with translocations were amplified with PCR using primers listed in Table S1. PCR conditions are 95°C 120 sec, 45 cycles of (95°C 10 sec, 62°C 10 sec, 72°C 40 sec) using TB Green Fast qPCR Mix and LightCycler Nano. Subsequently, PCR products were electrophoresed in an agarose gel and purified. DNA sequences were analyzed by FASMAC using primers listed in Table S1.

PCR condition for amplification of the CRISPR/Cas9 target *CCND1* site and sequencing

Genome DNA were amplified with PCR using primers listed in Table S1. PCR conditions are 95°C 120 sec, 45 cycles of (95°C 10 sec, 62°C 10 sec, 72°C 30 sec) using TB Green® Fast qPCR Mix and LightCycler Nano. Subsequently, PCR products were electrophoresed in an agarose gel and purified. DNA sequences were analyzed by FASMAC using primers listed in Table S1.

Establishment of BiPSCs with AID expression regulated by the doxycycline-controlled (Tet-off) system

The method was described in our previous study[3]. Briefly, AID cDNA was amplified from

pcDNA3.1AID (a kind gift of Dr. Yakushijin, Ehime University School of Medicine) with KOD plus DNA polymerase using the primers: hAID-BamHI and hAID-EcoRI. Amplified DNA was digested with BamHI and EcoRI, and cloned into the BamHI/EcoRI sites of pRetroX-Tight-Pur vector (Takara Bio) to form pRetroX-Tight-AID-Pur. The DNA sequence of the cloned AID fragment was confirmed. Next, packaged pRetroX-Tight-AID-Pur. was infected to pRetroX-TetOff Advanced-expressing BiPSC13 and MIB2-6 cells. Puromycin-resistant cell clones were picked and cultured in the presence of 10 ng/mL doxycycline (Takara Bio) to prevent AID expression.

qRT-PCR analysis of AID expression in G#28-AID and AX-AID induced by the doxycycline-controlled (Tet-off) system

Total RNA was isolated from cells using the RNeasy Mini Kit (QIAGEN, Germantown, MD, USA), treated with DNase I (Takara Bio), and first-strand cDNA was synthesized using ReverTra Ace reverse transcriptase (TOYOBO) according to the manufacturer's instructions. DNase I treatment and reverse transcription was performed using the LifeECO Thermal Cycler (BIOER Tech), and qRT-PCR was performed using Thunderbird SYBR qPCR Mix (TOYOBO) and the Light Cycler Nano (Roche) (n=3). Ratio of 2-ΔΔCq values were calculated from the values obtained by LightCycler Nano Software. The primers used for qRT-PCR are listed Table S1.

Detection of monoclonal *IgH* gene rearrangements

PCR analysis of *IgH* gene rearrangements (VH-JH and DH-JH) according to the

BIOMED-2 guidelines[2], using DNA extracted from BiPSCs, was performed at LSI Medience (LSI Medience Corporation, Tokyo, Japan).

Co-culture of BiPSCs with AGM-S3 for differentiation into Hematopoietic progenitor cells (HPCs)

The mouse AGM-S3 cell line (AGM)[4] was a gift from Kyowa Kirin Co., Ltd. (Tokyo, Japan) and was plated onto 10-cm dishes in α-MEM (Nakalai Tesque, Kyoto, Japan) supplemented with 10% fetal bovine serum (FBS) (Biowest, Nuaillé, France) and 1% penicillin/streptomycin (PS) (Nakalai Tesque) and cultured until about 80% confluent. BiPSCs were cultured on a matrigel (Matrigel hESC-Qualified Matrix)-coated FALCON tissue culture plate 6-well (Corning) in Complete StemFit AK02N for 5-7 days. Subsequently, BiPSCs were picked up as small clumps (30-50 pieces) equivalent to 1×10^5 cells and added to 20 Gy-irradiated AGM cultures in IMDM (Nakalai Tesque) supplemented with 10% FBS, 1% PS, 1% MEM non-essential amino acids solution (Nakalai Tesque), 0.5 mM 1-thioglycerol (Sigma-Aldrich), 1% L-ascorbic acid 2-phosphate sesquimagnesium salt hydrate (Tokyo Chemical Industry, Tokyo, Japan), 1% Insulin-Transferrin-Selenium (ITS -G) (Thermo Fisher Scientific KK), 1% GlutaMAX-I (Thermo Fisher Scientific KK), and 20 ng/mL human vascular endothelial growth factor (VEGF121) (Peprotech, Cranbury, NJ, USA). The BiPSC/AGM co-cultures were incubated at 37°C in normoxic conditions and 5% CO_2. The medium was replaced with new medium on days 3, 6, 9, and 13. On day 14, BiPSCs were resuspended into single cells with a 100-μm cell strainer (CORNING) after a 10-min incubation at 37°C with Accutase (Innovative Cell Technologies, San Diego, CA, USA).

Legends

Figure S1. B-cell differentiation from pro-B cell to plasma cell and representative cell surface antigens adapted from reference 5.

Pre-B cells express pre-BCR with heavy chain (HC) gene rearrangement. Differentiated B cells and plasma cells from pre-B cells express BCR, which is a complex of an HC and a light chain (LC) with gene rearrangement. BCR, B-cell receptor; SHM, somatic hypermutation.

Figure S2. Hypothesis of the mechanism by which myeloma-initiating cells are born.

If Yamanaka factors would be activated for any reasons in mature B cells, those cells would be reprogramed to transform to B cell-derived iPS cells (BiPSCs). During the redifferentiation of these BiPSCs into hematopoietic progenitor cells (HPCs) and further into B cells, DNA double-strand breaks (DSBs) would occur in chromosome 14 and another chromosome due to AID expression, resulting in the creation of myeloma-initiating cells with reciprocal chromosomal translocation (cTr) of these chromosomes. Furthermore, BiPSCs with cTr (t(11;14)) and *p53* deletion differentiate into HPCs, and gene mutations would be induced due to AID expression at the stage of differentiation into B cells, also resulting in the creation of myeloma-initiating cells. HSC, hematopoietic stem cell; AID, activation-induced cytidine deaminase.

Figure S3. Copy number analysis of the *IgH* constant region.

Each region of the *IgH* constant region (Iμ, IgM-Q, IgD-Q, IgG3-Q, and IgG1-Q) was amplified with PCR and compared with that of *CCND1* as a reference to analyze the defect status. Primers are listed in Supplemental Table 1. No decrease in copy number

was found in this region in BiPSC13, MIB2-6, and peripheral blood mononuclear cells (PBMNCs), which means that class switch recombination (CSR) has not occurred. The copy numbers of IgM-Q (Cμ) and IgD-Q (Cδ) were halved in EBV-infected B lymphocyte (C032), which means that CSR has occurred in one allele of *IgH*. No copies were found between IgM-Q and IgG3-Q in KMS-26 (myeloma cell line), which means that CSR to IgG1 has occurred in both alleles of *IgH*.

Figure S4. Nucleotide sequences of both alleles of *IgH* of BiPSC13 and MIB2-6.

(A) The VDJ region of the functional allele of *IgH* of BiPSC13 was consistent with the nucleotide sequence of $V_{3\text{-}9}D_{4\text{-}23}J_2$, and no somatic mutation was present in that region. N sequences were detected at the junctions between $V_{3\text{-}9}$ and $D_{4\text{-}23}$ and between $D_{4\text{-}23}$ and J_2. **(B)** The non-functional allele of *IgH* of BiPSC13 stopped at DJ rearrangement, and an N sequence was detected at the junction between $D_{2\text{-}21}$ and J_2. **(C)** The VDJ region of the functional allele of *IgH* of MIB2-6 was consistent with the nucleotide sequence of $V_{4\text{-}39}D_{3\text{-}22}J_6$, and no somatic mutation was derected in that region. N sequences were detected at the junctions between $V_{4\text{-}39}$ and $D_{3\text{-}22}$ and between $D_{3\text{-}22}$ and J_6. **(D)** The non-functional allele of *IgH* of MIB2-6 was suspected to have ended imperfectly with a gap of more than 1000 bases between $D_{5\text{-}18}$ and J_4.

Figure S5. Effects of CRISPR/Cas9 on alleles of *IgH* not used in the translocation with *CCND1*.

(A) The cleavage sites of Cas9 in the vicinity of Eμ (between Eμ-Cμ) of *IgH* of BiPSC13 and MIB2-6 are shown. Both alleles showed no mutation in this region compared with the germline sequence. **(B)** The repair process after cleavage by Cas9 revealed a deletion of 14 bases in the DJ rearrangement allele of AZ, and a deletion of one base in the VDJ

rearrangement allele of AX, a deletion of 12 bases in the DJ rearrangement allele of BC and a deletion of 28 bases in the VDJ rearrangement allele of BG.

Figure S6. Effects of CRISPR/Cas9 on alleles of *CCND1* not used in the translocation with *IgH*.

(A) The cleavage sites of Cas9 upstream of the protein-coding sequence of *CCND1* are shown. No mutation was present in this region compared with the germline sequence (B). **(B)** The repair process after cleavage by Cas9 revealed a deletion of 11 bases in AZ, a deletion of 11 bases in AX, a deletion of 11 bases in BG, and a deletion of 333 bases in BC.

Figure S7. qRT-PCR and western blot analysis of AID expression in G#28-AID and AX-AID induced by the doxycycline-controlled (Tet-off) system.

(A) AID expression in G#28-AID and AX-AID in the absence of doxycycline was measured using qRT-PCR. The numbers on the Y axis are the expression of AID mRNA normalized to the expression of GAPDH relative to the expression of AID mRNA of $CD19^+$ normal B cells. Data were analyzed in triplicate. (B) AID expression as seen with by western blotting.

Figure S8. Induction of differentiation of BiPSC13 and MIB2-6 into HPCs

Flow cytometric analysis of the cell phenotype after differentiation of BiPSC13 (A) and MIB2-6 (B) into HPCs. $CD34^+$ cells were mainly $CD43^-$/$CD45^-$/$CD38^-$.

Figure S9. Phenotype analysis of purified BiPSC13-derived $CD34^+$ cells

Purified CD34+ cells were evaluated by two-color and three-color flow cytometry after staining with the following antibodies: FITC mouse IgG1, κisotype Ctrl antibody (BioLegend), PE mouse IgG1, κisotype Ctrl antibody (BioLegend), and PE/Cyanine5 mouse IgG1, κisotype Ctrl antibody (BioLegend). Cell populations circled with red dotted lines emitted autofluorescence. Phenotype analysis of mixed floating and adherent cells 3 weeks (A) and 5 weeks (B) weeks after initiating the co-culture of BiPSC13 and MS-5.

Figure S10. Nucleotide sequences of both alleles of *IgH* of two myeloma cell lines, NOP-2[6] and KMS-12[7].

(A) Monoclonal VDJ rearrangements of *IgH* (arrows) in NOP-2 detected using PCR. The arrow with # indicates that the height of the peak is less than that of the positive control in FR3-JH. **(B)** The VDJ region of the functional allele of *IgH* of NOP-2 was consistent with the nucleotide sequence of $V_{1\text{-}46}D_{5\text{-}18}J_6$, and several somatic mutations were present in that region. Red circles indicate amino acid substitutions. N sequences were detected at the junctions between $V_{1\text{-}46}$ and $D_{5\text{-}18}$ and between $D_{5\text{-}18}$ and J_6. **(C)** The non-functional allele of the *IgH* of NOP-2 stopped at DJ rearrangement, and an N sequence was detected at the junction between $D_{2\text{-}21}$ and J_5. **(D)** Monoclonal VDJ rearrangements of *IgH* (arrows) in KMS-12 detected using PCR. The arrow with # indicates that the height of the peak is less than that of the positive control in FR2-JH. **(E)** The VDJ region of the functional allele of *IgH* of KMS-12 was consistent with the nucleotide sequence of $V_{3\text{-}7}D_{4\text{-}23}J_6$, and several somatic mutations were present in that region. Red circles indicate amino acid substitutions. N sequences were detected at the junctions between $V_{3\text{-}7}$ and $D_{4\text{-}23}$ and between $D_{4\text{-}23}$ and J_6. **(F)** The non-functional allele of *IgH* of KMS-12 stopped at DJ rearrangement, and an N sequence was detected at the junction between $D_{6\text{-}25}$ and J_4.

References

1. Livak KJ, Schmittgen TD. Analysis of relative gene expression data using real-time quantitative PCR and the 2(-Delta Delta C(T)) Method. *Methods* 25:402-408(2001).
2. van Dongen, J. J. M. *et al.* Design and standardization of PCR primers and protocols for detection of clonal immunoglobulin and T-cell receptor gene recombinations in suspect lymphoproliferations: Report of the BIOMED-2 concerted action BMH4-CT98-3936. *Leukemia* **17**, 2257–2317 (2003).
3. Kawamura, F. *et al.* Establishment of induced pluripotent stem cells from normal B cells and inducing AID expression in their differentiation into hematopoietic progenitor cells. *Sci. Rep.* **7**, 1–11 (2017).
4. Xu, M. J. *et al.* Stimulation of mouse and human primitive hematopoiesis by murine embryonic aorta-gonad-mesonephros-derived stromal cell lines. *Blood* **92**, 2032–2040 (1998).
5. Jaffe ES, *et al.* Introduction and overview of the classification of lymphoid neoplasms. in *WHO classification of tumours of haematopoietic and lymphoid tissues*. (eds. Swerdlow SH, *et al.*) 190-198 (International Agency for Research on Cancer, 2017).
6. Nagai, T. *et al.* Establishment and characterization of a new human Bence Jones-type myeloma cell line, NOP-2. *Int. J. Hematol.* **54** , 141–149(1991).
7. Ohtsuki, T. *et al.* Two human myeloma cell lines, amylase-producing KMS-12-PE and amylase-non-producing KMS-12-BM, were established from a patient, having the same chromosome marker, t(11;14)(q13;q32). *Bri. J. Haematol.* **73**, 199–204(1989).

Table S1. Primers for PCR

IgH V region primers to confirm VDJ or DJ rearrangement

VH1-FR1	GGCCTCAGTGAAGGTCTCCTGCAAG
VH2-FR1	GTCTGGTCCTACGCTGGTGAAACCC
VH3-FR1	CTGGGGGGTCCCTGAGACTCTCCTG
VH4-FR1	CTTCGGAGACCCTGTCCCTCACCTG
VH5-FR1	CGGGGAGTCTCTGAAGATCTCCTGT
VH6-FR1	TCGCAGACCCTCTCACTCACCTGTG

IgH D region primers to confirm VDJ or DJ rearrangement (5' to 3')

DH1	GGCGGAATGTGTGCAGGC
DH2	GCACTGGGCTCAGAGTCCTCT
DH3	GTGGCCCTGGGAATATAAAA
DH4	AGATCCCCAGGACGCAGCA
DH5	CAGGGGGACACTGTGCATGT
DH6	TGACCCCAGCAAGGGAAGG
DH7	CACAGGCCCCCTACCAGC

IgH J primer to confirm VDJ or DJ rearrangement (5' to 3')

JH consensus	CTTACCTGAGGAGACGGTGACC

Primers to comfirm t(11;14) [#1] (5' to 3')

IgH-Fs[#2]	AAGGGTGCGATGATGACCTAC
IgHμ-LoF[#3]	ATTTCCACTAGAAGGGGAACTGGTCTTAAT
DH5-18-F	CCTACACCAGAGCCAGCAAAG
CCND1-86-F[#4]	CTTCTCACGAGCTGCCTTTG
CCND1-86-R	GCTCATCACACAGCTTGACG
CCND1-86-Rs	AGCTGTTCTTGTAGTGGTGCC

Primers to analyze copy number of *CCND1* and *IgH* constant region (5' to 3')

CCND1-86-F	CTTCTCACGAGCTGCCTTTG
CCND1-86-Rt	GCCTACCACACCTCTTTTCCA
IgH-F	TTAGACAAGGGCGATGCCAG
IgH-R3	CCACTAGAAGGGGAACTGGTC
IgM-Q-F	ACCGTGTCCGAAGAGGAATG
IgM-Q-R	TGGGTTTACCGGTGGACTTG
IgD-Q-F	GTACCACCCAACGTCCGTGA
IgD-Q-R	GATCTCCGGTGCGACCTACC
IgG3-Q-F	GTCCCCACCTGACACTATCTTCTGT
IgG3-Q-R	AAGATCCACTTCACCTGTAGGCA
IgG1-Q-F	CCTGTATGAAACCCTGTCCCAC
IgG1-Q-R	CGGGTTTAGGTAAGAACAACGTG

Primers to clone AID open reading frame

hAID-BamHI	TGGGATCCGCCACCATGGAC
hAID-EcoRI	CAGAATTCTCAAAGTCCCAAAGTACG

Primers to analyze AID expression by qRT-PCR (5' to 3')

AID-F	AAAATGTCCGCTGGGCTAAG
AID-R	AGGTCCCAGTCCGAGATGTAG
GAPDH-F	GGTGAAGGTCGGAGTCAACG
GAPDH-R	AATTTGCCATGGGTGGAATC

[#1] Primers were positioned in translocation junction site.
[#2] Primer for DNA sequence analysis of der(14)t(11;14)
[#3] Primer for DNA sequence analysis of CRISPR/Cas9 target *IgH* site

[#4] Primer for DNA sequence analysis of der(11)t(11;14) and CRISPR/Cas9 target *CCND1* site

Figure S1

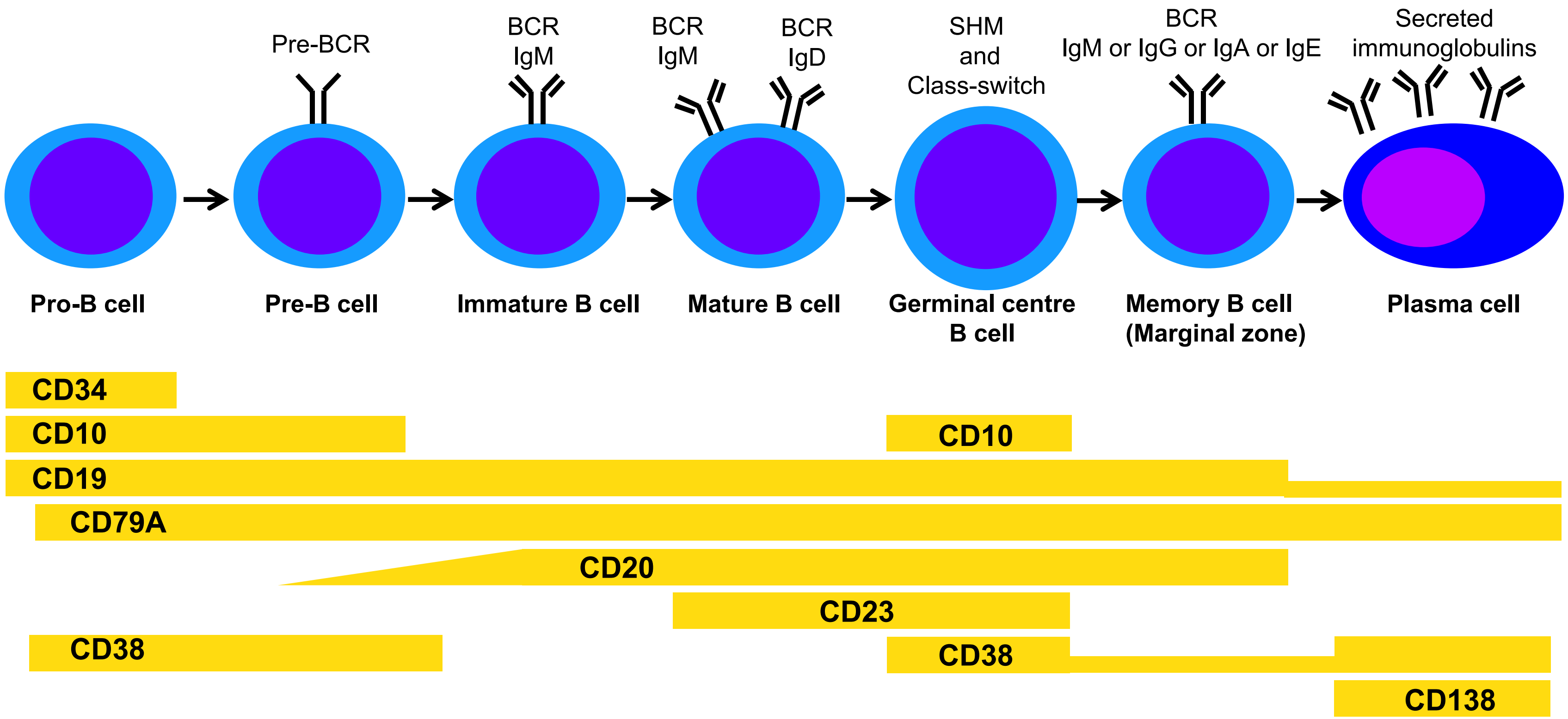

Pre-BCR
BCR
IgM
BCR
IgM
BCR
IgD
SHM
and
Class-switch
BCR
IgM or IgG or IgA or IgE
Secreted
immunoglobulins
Pro-B cell
Pre-B cell
Immature B cell
Mature B cell
Germinal centre
B cell
Memory B cell
(Marginal zone)
Plasma cell
CD34
CD10
CD10
CD19
CD79A
CD20
CD23
CD38
CD38
CD138

Figure S2

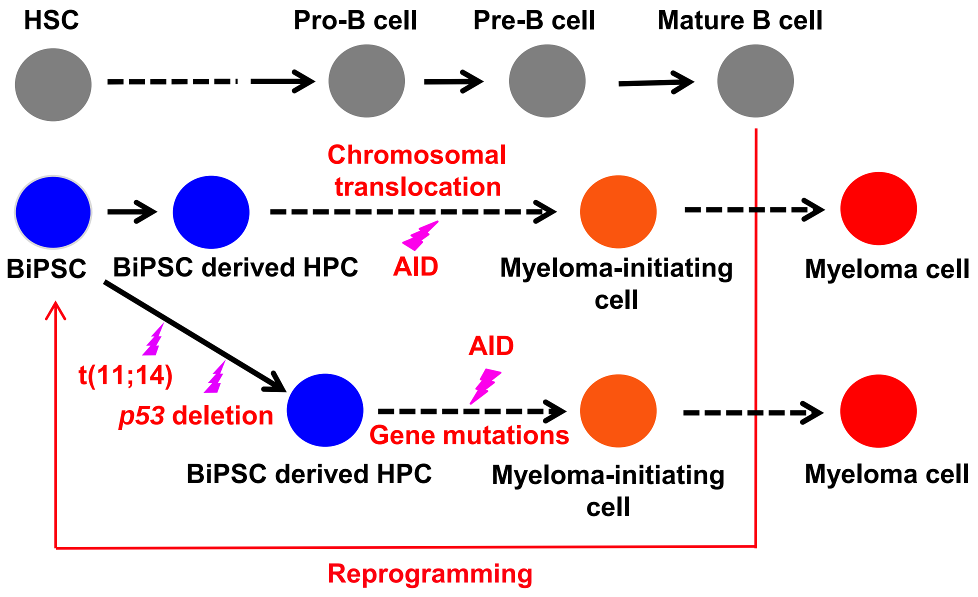

HSC
Pro-B cell
Pre-B cell
Mature B cell
Chromosomal translocation
BiPSC
BiPSC derived HPC
AID
Myeloma-initiating cell
Myeloma cell
AID
t(11;14)
p53 deletion
Gene mutations
BiPSC derived HPC
Myeloma-initiating cell
Myeloma cell
Reprogramming

Figure S3

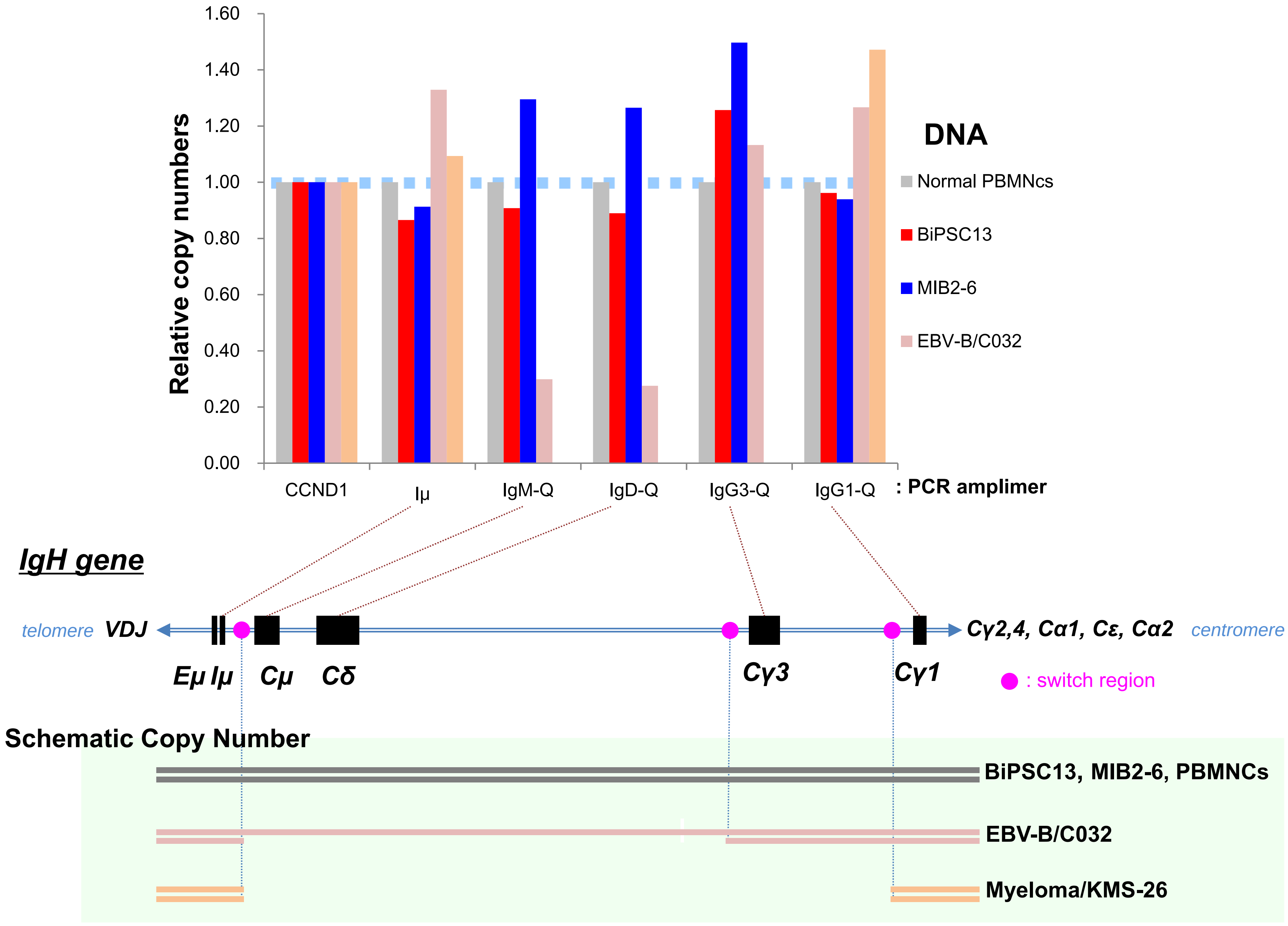

1.60
1.40
1.20
1.00
0.80
0.60
0.40
0.20
0.00
Relative copy numbers
CCND1
Iμ
IgM-Q
IgD-Q
IgG3-Q
IgG1-Q
: PCR amplimer
DNA
Normal PBMNcs
BiPSC13
MIB2-6
EBV-B/C032
IgH gene
telomere
VDJ
Eμ Iμ
Cμ
Cδ
Cγ3
Cγ1
Cγ2,4, Cα1, Cε, Cα2
centromere
: switch region
Schematic Copy Number
BiPSC13, MIB2-6, PBMNCs
EBV-B/C032
Myeloma/KMS-26

Figure S4

A

```
                                 V3-9
                A  A  S  G  F  T  F  D  D  Y  A  M  H  W  V  R  Q  A  P  G  K  G  L  E  W  V  S  G  I  S
BiPSC13      1  TGCAGCCTCTGGATTCACCTTTGATGATTATGCCATGCACTGGGTCCGGCAAGCTCCAGGGAAGGGCCTGGAGTGGGTCTCAGGTATTAG  90
IGHV3-9*01  66  ..........................................................................................  155
                A  A  S  G  F  T  F  D  D  Y  A  M  H  W  V  R  Q  A  P  G  K  G  L  E  W  V  S  G  I  S

                W  N  S  G  S  I  G  Y  A  D  S  V  K  G  R  F  T  I  S  R  D  N  A  K  N  S  L  Y  L  Q
BiPSC13     91  TTGGAATAGTGGTAGCATAGGCTATGCGGACTCTGTGAAGGGCCGATTCACCATCTCCAGAGACAACGCCAAGAACTCCCTGTATCTGCA  180
IGHV3-9*01 156  ..........................................................................................  245
                W  N  S  G  S  I  G  Y  A  D  S  V  K  G  R  F  T  I  S  R  D  N  A  K  N  S  L  Y  L  Q

                                                                  D4-23                   J2
                M  N  S  L  R  A  E  D  T  A  L  Y  Y  C  A  K  A  V  A  H  P  Y  W  Y  F  D  L  W  G  R
BiPSC13    181  AATGAACAGTCTGAGAGCTGAGGACACGGCCTTGTATTACTGTGCAAAGGCGGTGGCCCACCCCTACTGGTACTTCGATCTCTGGGGCCG  270
IGHV3-9*01 246  ................................................------------------------------------------  293
                M  N  S  L  R  A  E  D  T  A  L  Y  Y  C  A
IGHD4-23*01  7  --------------------------------------------------......----------------------------------  12
IGHJ2*01     1  ---------------------------------------------------------------...........................  27

                G  T  L
BiPSC13    271  TGGCACCCT  279
IGHJ2*01    28  .........  36
```

Figure S4

B

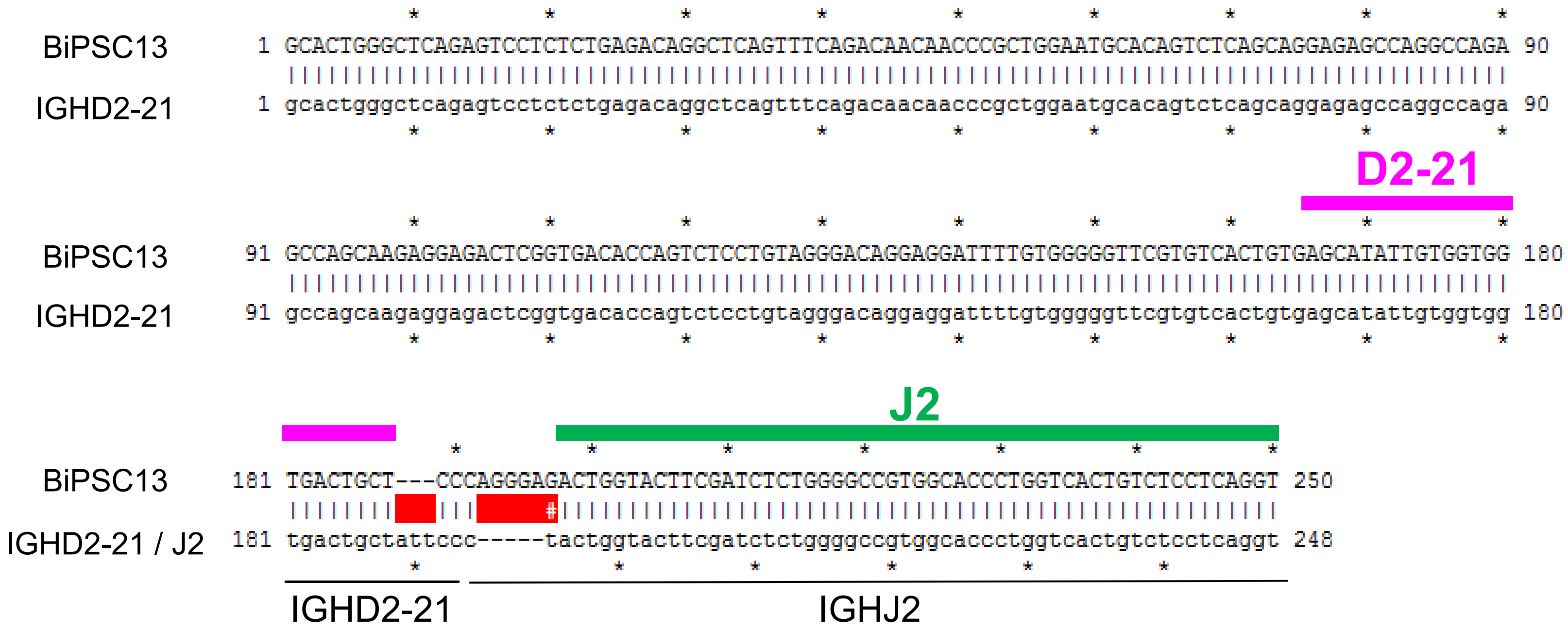
BiPSC13
1 GCACTGGGCTCAGAGTCCTCTCTGAGACAGGCTCAGTTTCAGACAACAACCCGCTGGAATGCACAGTCTCAGCAGGAGAGCCAGGCCAGA 90
IGHD2-21
1 gcactgggctcagagtcctctctgagacaggctcagtttcagacaacaacccgctggaatgcacagtctcagcaggagagccaggccaga 90
D2-21
BiPSC13
91 GCCAGCAAGAGGAGACTCGGTGACACCAGTCTCCTGTAGGGACAGGAGGATTTTGTGGGGGTTCGTGTCACTGTGAGCATATTGTGGTGG 180
IGHD2-21
91 gccagcaagaggagactcggtgacaccagtctcctgtagggacaggaggattttgtgggggttcgtgtcactgtgagcatattgtggtgg 180
J2
BiPSC13
181 TGACTGCT---CCCAGGGAGACTGGTACTTCGATCTCTGGGGCCGTGGCACCCTGGTCACTGTCTCCTCAGGT 250
IGHD2-21 / J2
181 tgactgctattccc-----tactggtacttcgatctctggggccgtggcaccctggtcactgtctcctcaggt 248
IGHD2-21
IGHJ2

Figure S4

C

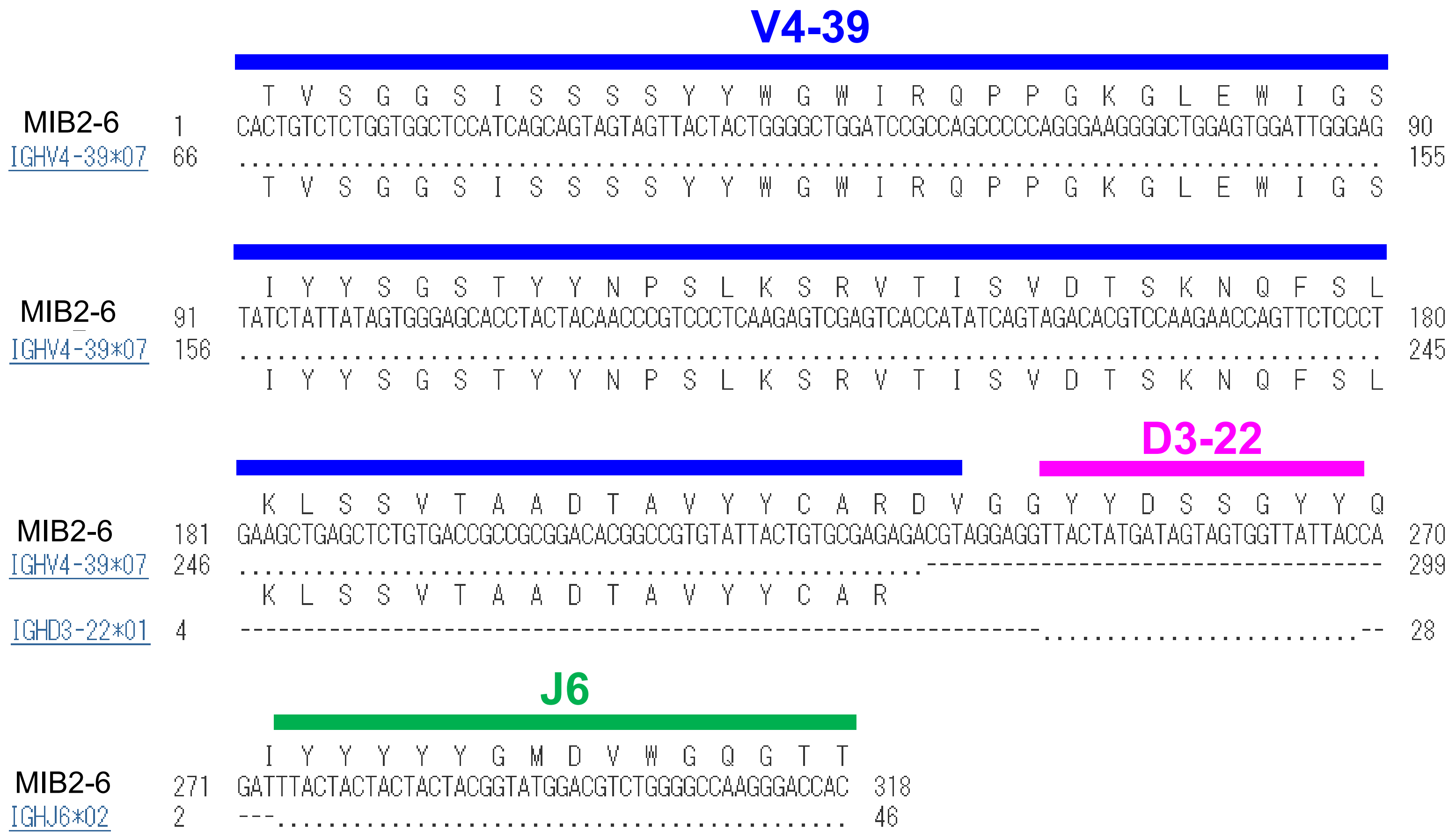

V4-39
T V S G G S I S S S S Y Y W G W I R Q P P G K G L E W I G S
MIB2-6 1 CACTGTCTCTGGTGGCTCCATCAGCAGTAGTAGTTACTACTGGGGCTGGATCCGCCAGCCCCCAGGGAAGGGGCTGGAGTGGATTGGGAG 90
IGHV4-39*07 66 155
T V S G G S I S S S S Y Y W G W I R Q P P G K G L E W I G S
I Y Y S G S T Y Y N P S L K S R V T I S V D T S K N Q F S L
MIB2-6 91 TATCTATTATAGTGGGAGCACCTACTACAACCCGTCCCTCAAGAGTCGAGTCACCATATCAGTAGACACGTCCAAGAACCAGTTCTCCCT 180
IGHV4-39*07 156 245
I Y Y S G S T Y Y N P S L K S R V T I S V D T S K N Q F S L
D3-22
K L S S V T A A D T A V Y Y C A R D V G G Y Y D S S G Y Y Q
MIB2-6 181 GAAGCTGAGCTCTGTGACCGCCGCGGACACGGCCGTGTATTACTGTGCGAGAGACGTAGGAGGTTACTATGATAGTAGTGGTTATTACCA 270
IGHV4-39*07 246 299
K L S S V T A A D T A V Y Y C A R
IGHD3-22*01 4 28
J6
I Y Y Y Y Y G M D V W G Q G T T
MIB2-6 271 GATTTACTACTACTACTACGGTATGGACGTCTGGGGCCAAGGGACCAC 318
IGHJ6*02 2 46

Figure S4

D

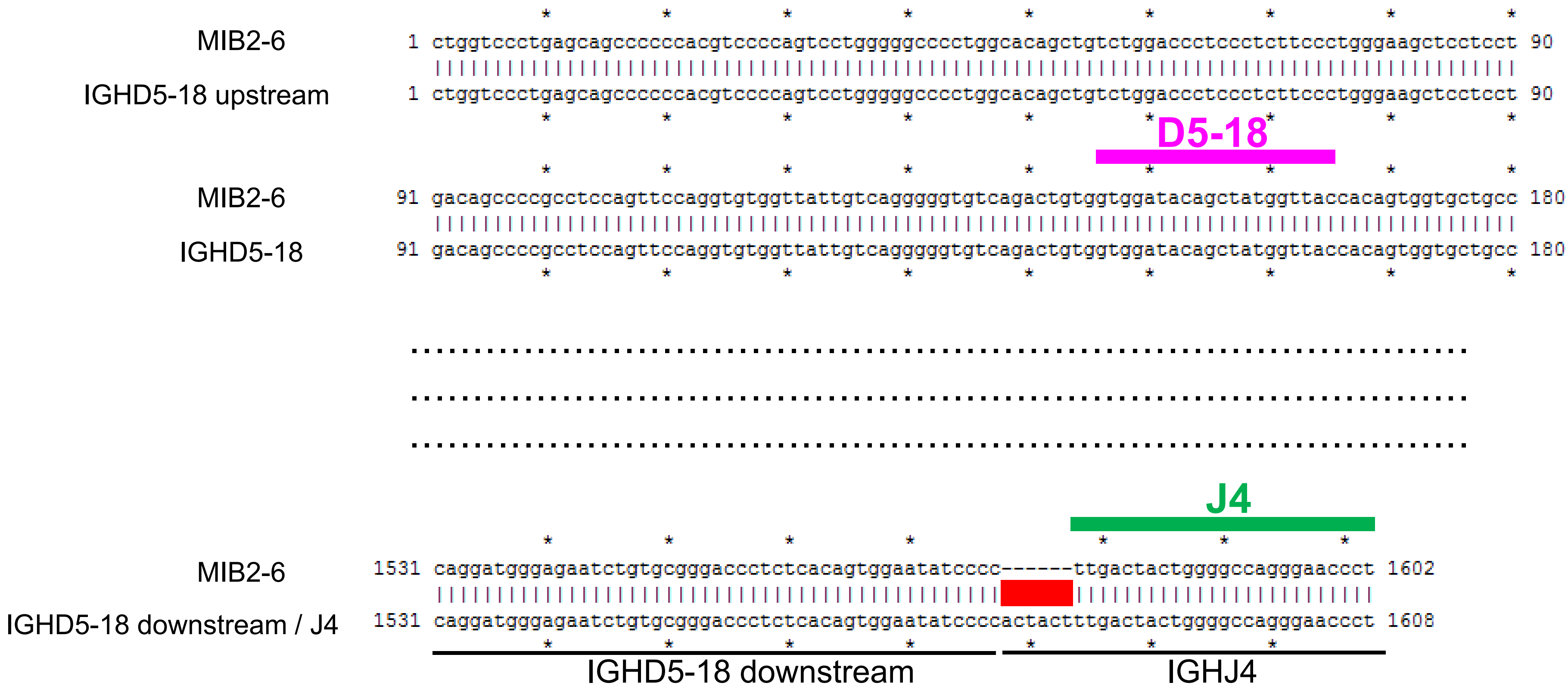
MIB2-6
1 ctggtccctgagcagcccccacgtccccagtcctggggcccctggcacagctgtctggaccctccctcttccctgggaagctcctcct 90
IGHD5-18 upstream
1 ctggtccctgagcagcccccacgtccccagtcctggggcccctggcacagctgtctggaccctccctcttccctgggaagctcctcct 90
D5-18
MIB2-6
91 gacagccccgcctccagttccaggtgtggttattgtcaggggtgtcagactgtggtggatacagctatggttaccacagtggtgctgcc 180
IGHD5-18
91 gacagccccgcctccagttccaggtgtggttattgtcaggggtgtcagactgtggtggatacagctatggttaccacagtggtgctgcc 180
J4
MIB2-6
1531 caggatgggagaatctgtgcgggaccctctcacagtggaatatcccc------ttgactactggggccagggaaccct 1602
IGHD5-18 downstream / J4
1531 caggatgggagaatctgtgcgggaccctctcacagtggaatatccccactactttgactactggggccagggaaccct 1608
IGHD5-18 downstream
IGHJ4

Figure S5

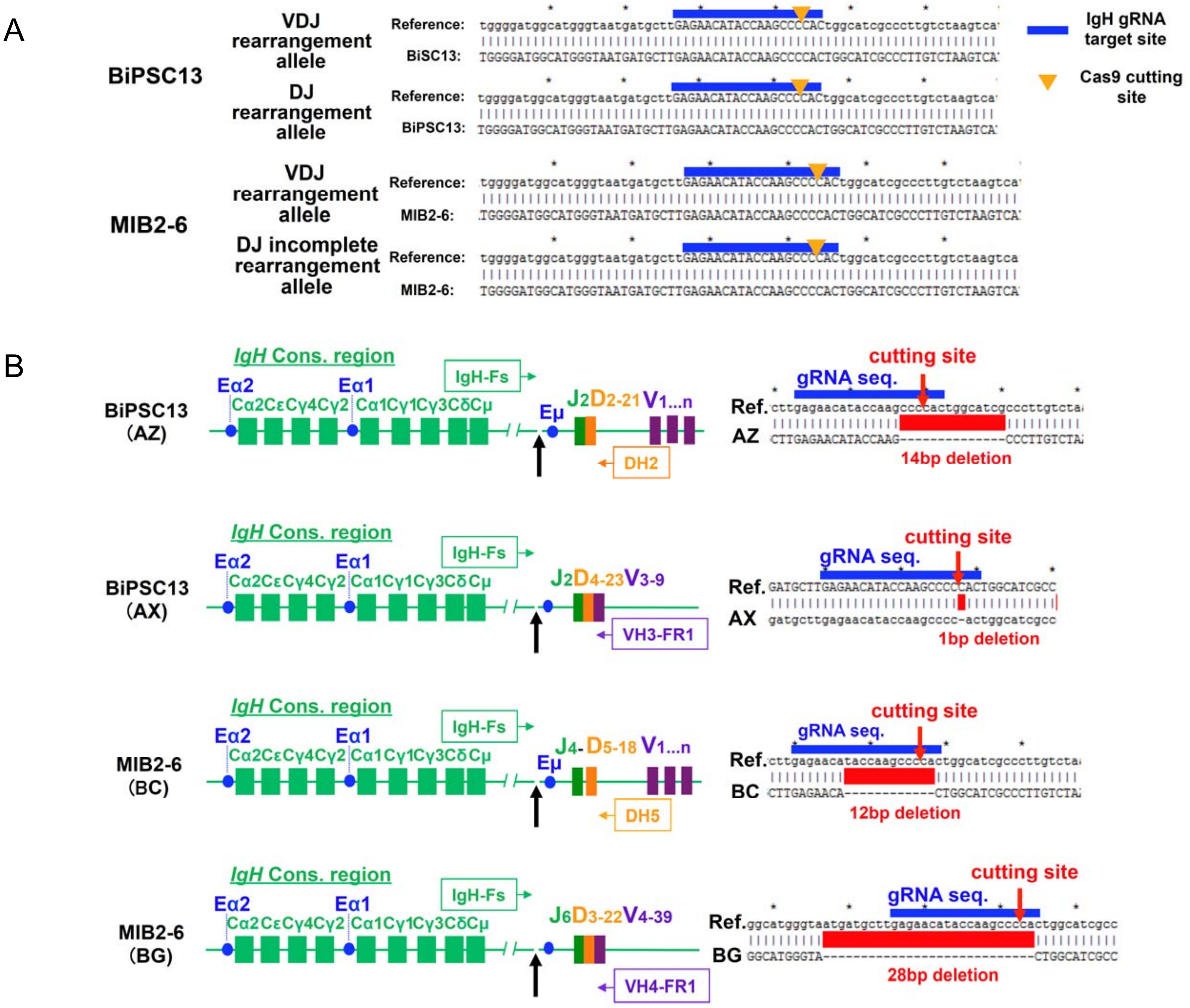
A
BiPSC13
VDJ rearrangement allele
Reference: tggggatggcatgggtaatgatgcttGAGAACATACCAAGCCCCACtggcatcgcccttgtctaagtca
BiSC13: TGGGGATGGCATGGGTAATGATGCTTGAGAACATACCAAGCCCCACTGGCATCGCCCTTGTCTAAGTCA
DJ rearrangement allele
Reference: tggggatggcatgggtaatgatgcttGAGAACATACCAAGCCCCACtggcatcgcccttgtctaagtca
BiPSC13: TGGGGATGGCATGGGTAATGATGCTTGAGAACATACCAAGCCCCACTGGCATCGCCCTTGTCTAAGTCA
MIB2-6
VDJ rearrangement allele
Reference: tggggatggcatgggtaatgatgcttGAGAACATACCAAGCCCCACtggcatcgcccttgtctaagtca
MIB2-6: TGGGGATGGCATGGGTAATGATGCTTGAGAACATACCAAGCCCCACTGGCATCGCCCTTGTCTAAGTCA
DJ incomplete rearrangement allele
Reference: tggggatggcatgggtaatgatgcttGAGAACATACCAAGCCCCACtggcatcgcccttgtctaagtca
MIB2-6: TGGGGATGGCATGGGTAATGATGCTTGAGAACATACCAAGCCCCACTGGCATCGCCCTTGTCTAAGTCA
IgH gRNA target site
Cas9 cutting site
B
BiPSC13 (AZ)
IgH Cons. region
IgH-Fs
Eα2
Eα1
Cα2CεCγ4Cγ2
Cα1Cγ1Cγ3CδCμ
Eμ
J2D2-21V1...n
DH2
cutting site
gRNA seq.
Ref. cttgagaacataccaagccccactggcatcgcccttgtcta
AZ CTTGAGAACATACCAAG--------------CCCTTGTCTA
14bp deletion
BiPSC13 (AX)
IgH Cons. region
IgH-Fs
Eα2
Eα1
Cα2CεCγ4Cγ2
Cα1Cγ1Cγ3CδCμ
J2D4-23V3-9
VH3-FR1
cutting site
gRNA seq.
Ref. GATGCTTGAGAACATACCAAGCCCCACTGGCATCGCC
AX gatgcttgagaacataccaagcccc-actggcatcgcc
1bp deletion
MIB2-6 (BC)
IgH Cons. region
IgH-Fs
Eα2
Eα1
Cα2CεCγ4Cγ2
Cα1Cγ1Cγ3CδCμ
Eμ
J4-D5-18V1...n
DH5
cutting site
gRNA seq.
Ref. cttgagaacataccaagccccactggcatcgcccttgtcta
BC CTTGAGAACA------------CTGGCATCGCCCTTGTCTA
12bp deletion
MIB2-6 (BG)
IgH Cons. region
IgH-Fs
Eα2
Eα1
Cα2CεCγ4Cγ2
Cα1Cγ1Cγ3CδCμ
J6D3-22V4-39
VH4-FR1
cutting site
gRNA seq.
Ref. ggcatgggtaatgatgcttgagaacataccaagccccactggcatcgcc
BG GGCATGGGTA----------------------------CTGGCATCGCC
28bp deletion

Figure S6

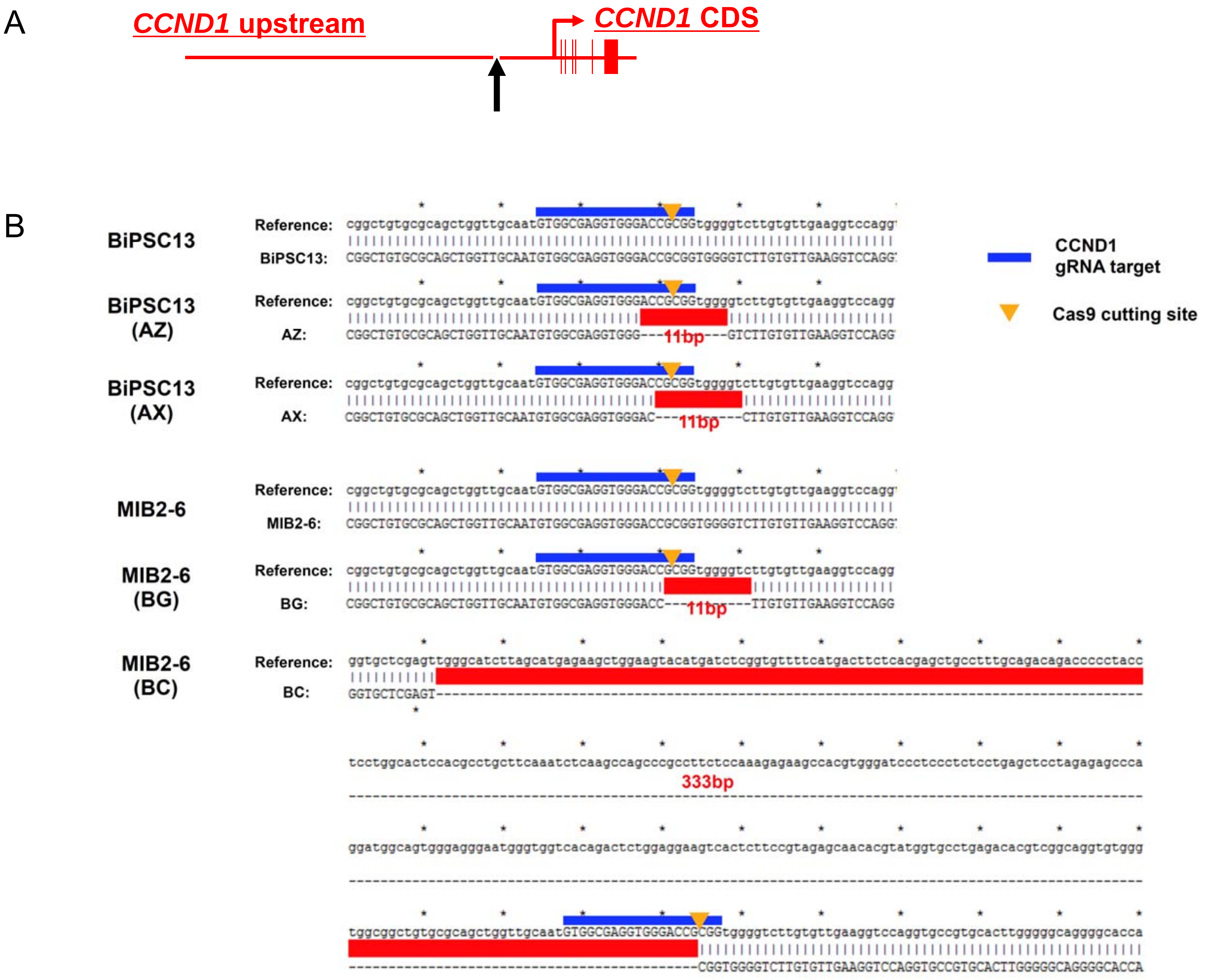
A
CCND1 upstream
CCND1 CDS
B
BiPSC13
BiPSC13 (AZ)
BiPSC13 (AX)
MIB2-6
MIB2-6 (BG)
MIB2-6 (BC)
Reference:
BiPSC13:
AZ:
AX:
MIB2-6:
BG:
BC:
11bp
333bp
CCND1 gRNA target
Cas9 cutting site

Figure S7

A

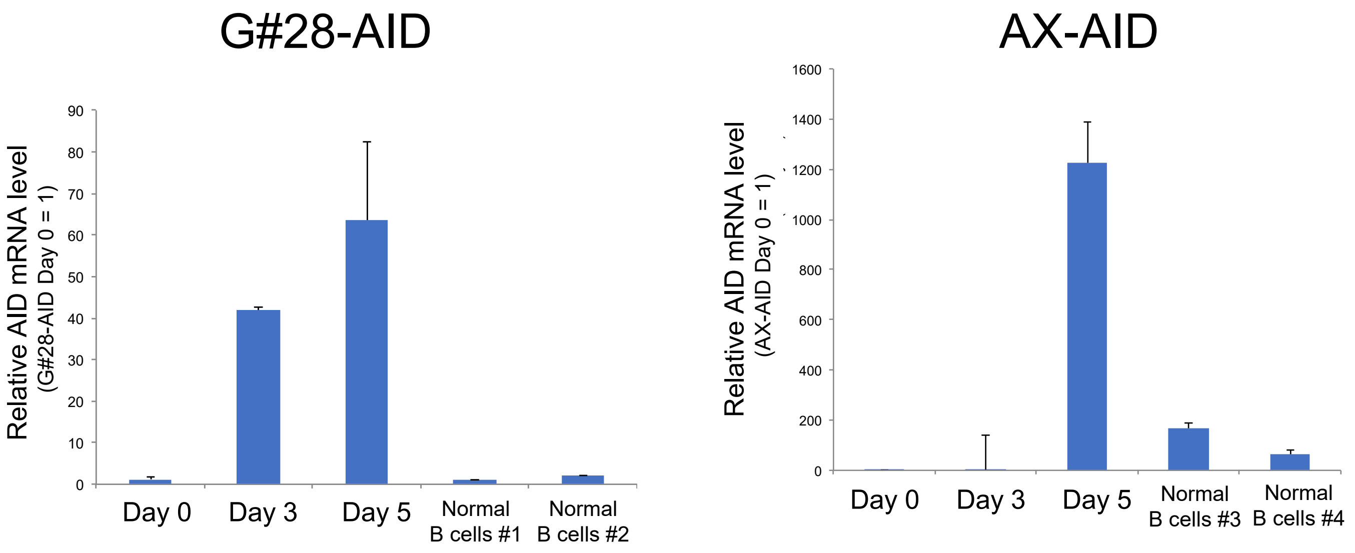
G#28-AID
Relative AID mRNA level
(G#28-AID Day 0 = 1)
90
80
70
60
50
40
30
20
10
0
Day 0
Day 3
Day 5
Normal B cells #1
Normal B cells #2
AX-AID
Relative AID mRNA level
(AX-AID Day 0 = 1)
1600
1400
1200
1000
800
600
400
200
0
Day 0
Day 3
Day 5
Normal B cells #3
Normal B cells #4

B

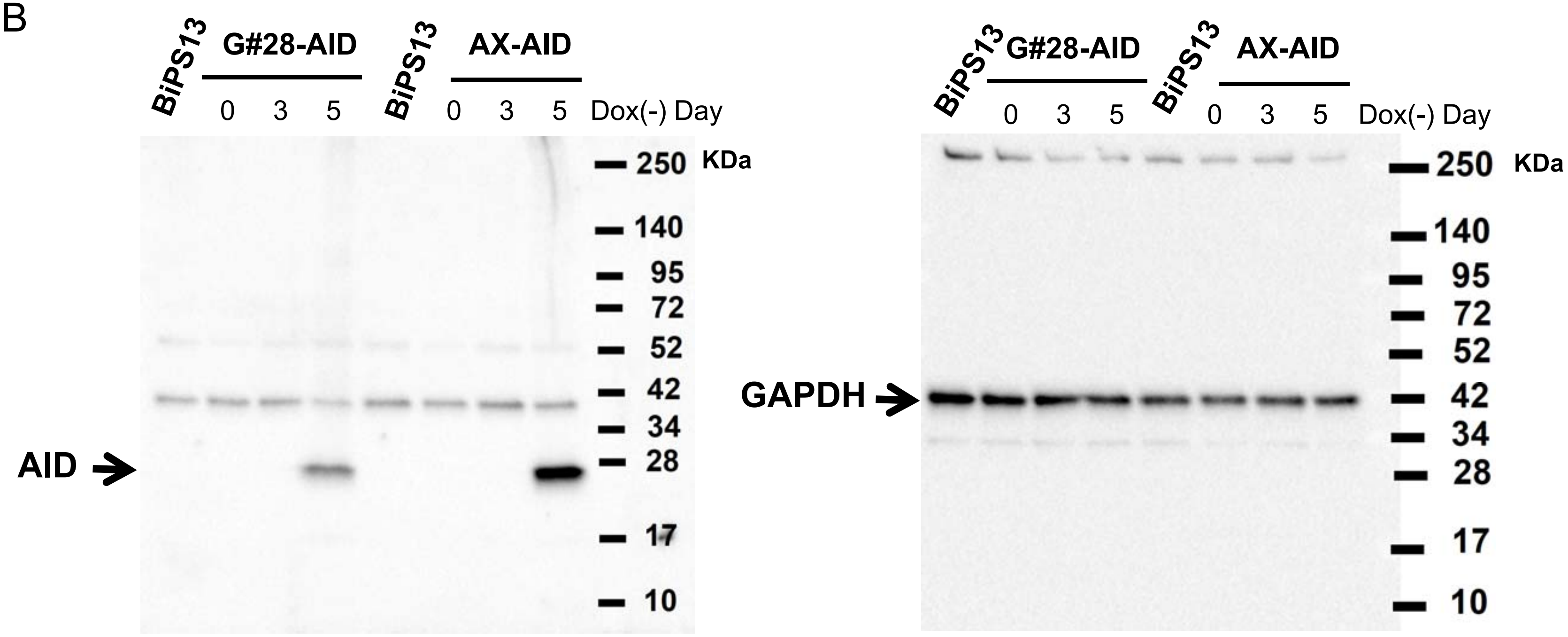
BiPS13
G#28-AID
BiPS13
AX-AID
0
3
5
0
3
5
Dox(-) Day
250
KDa
140
95
72
52
42
34
28
17
10
AID
GAPDH

Figure S8

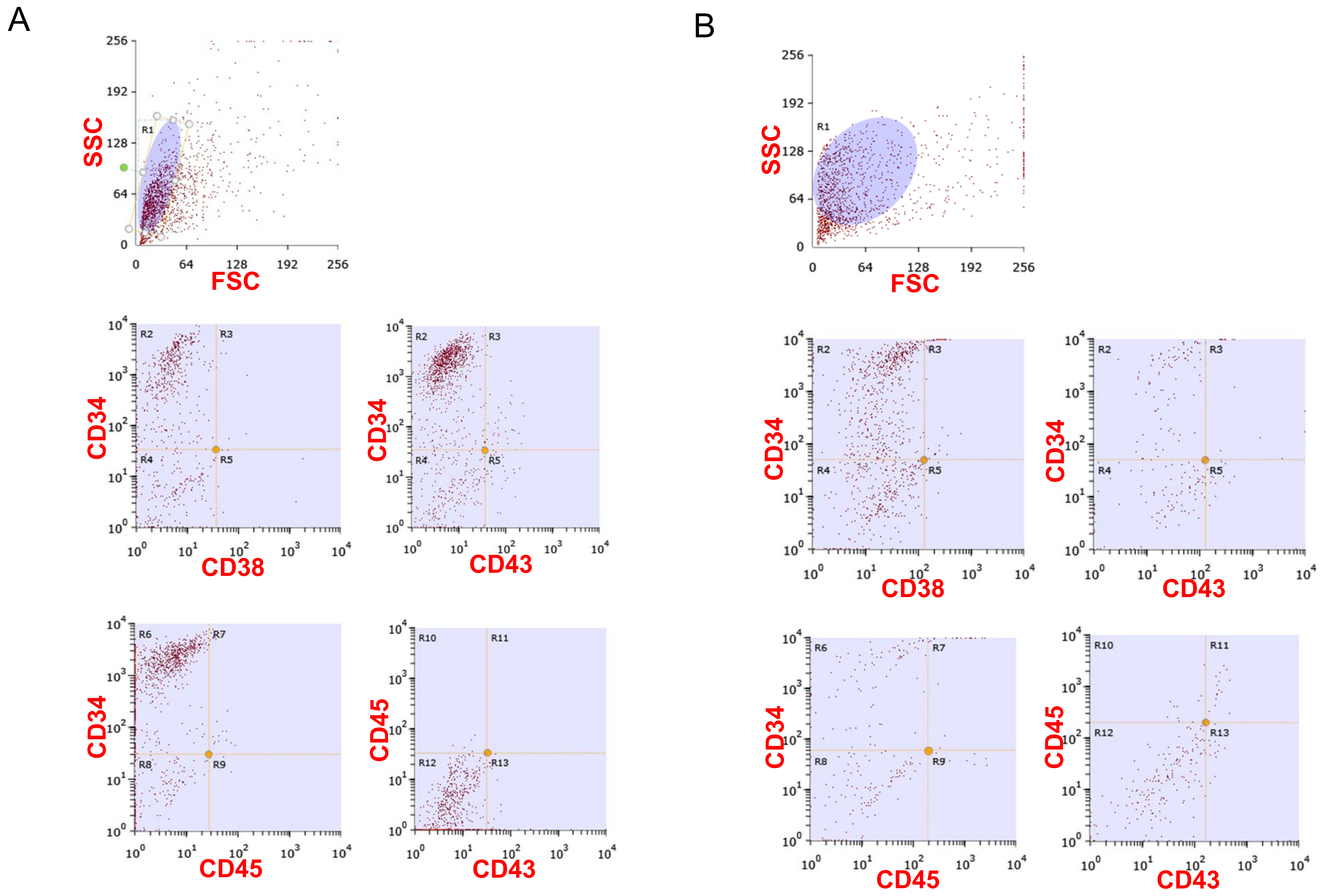
A
B
SSC
FSC
R1
CD34
CD38
CD43
CD45
R2
R3
R4
R5
R6
R7
R8
R9
R10
R11
R12
R13

Figure S9

A BiPSC13/MS-5 (3W)

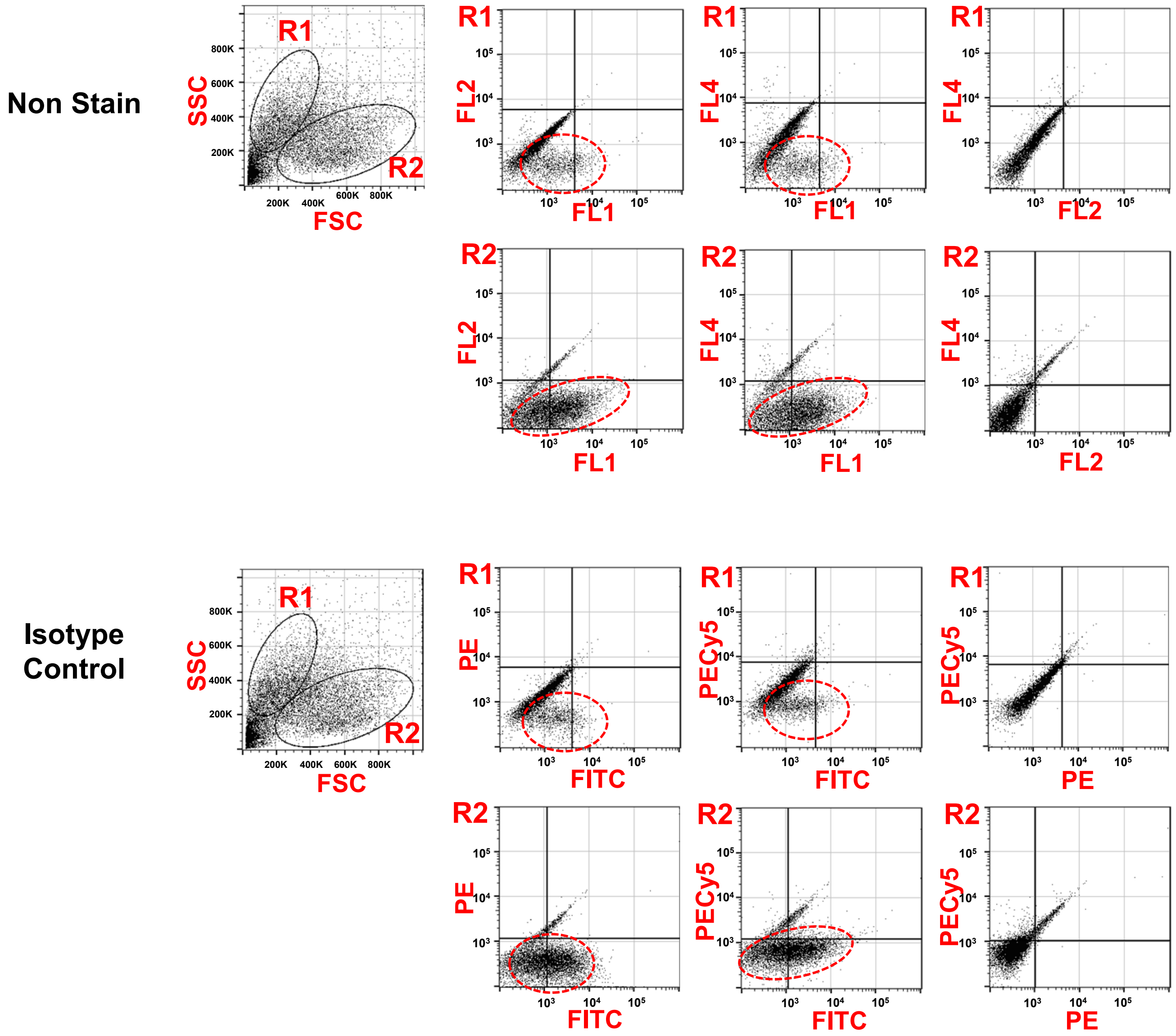

Figure S9

B BiPSC13/MS-5 (5W)

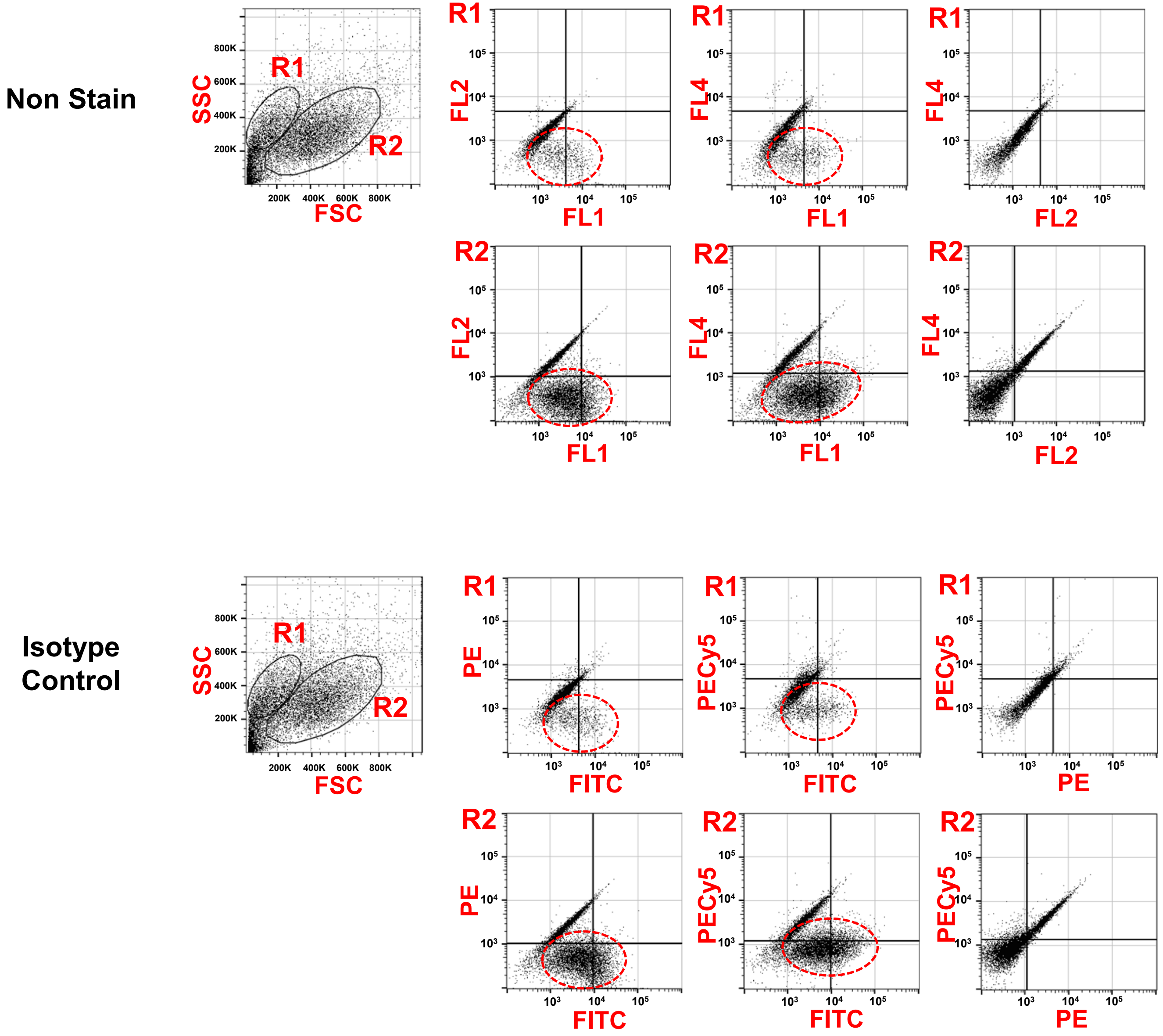

Figure S10

A NOP-2

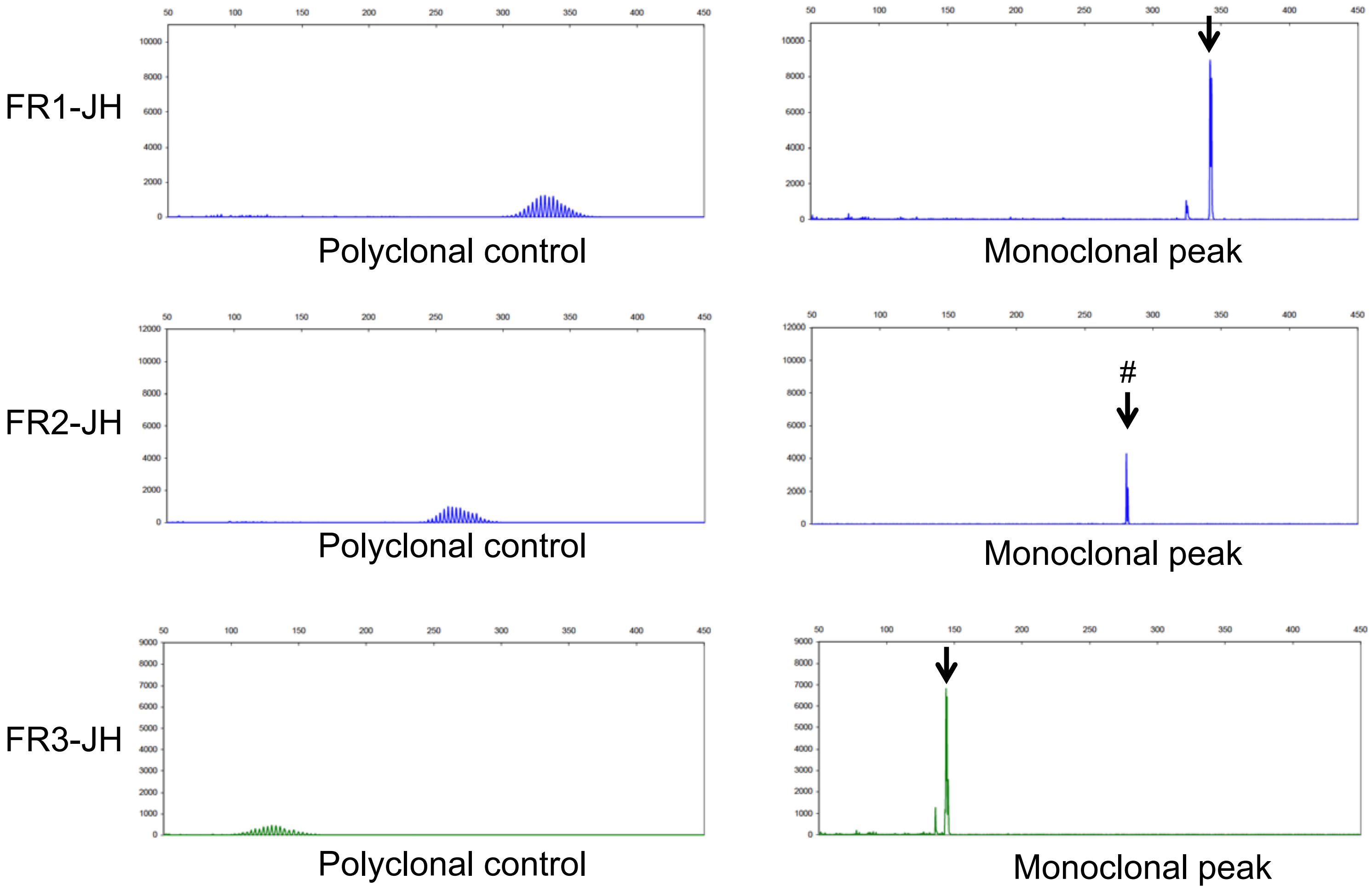

Figure S10

B

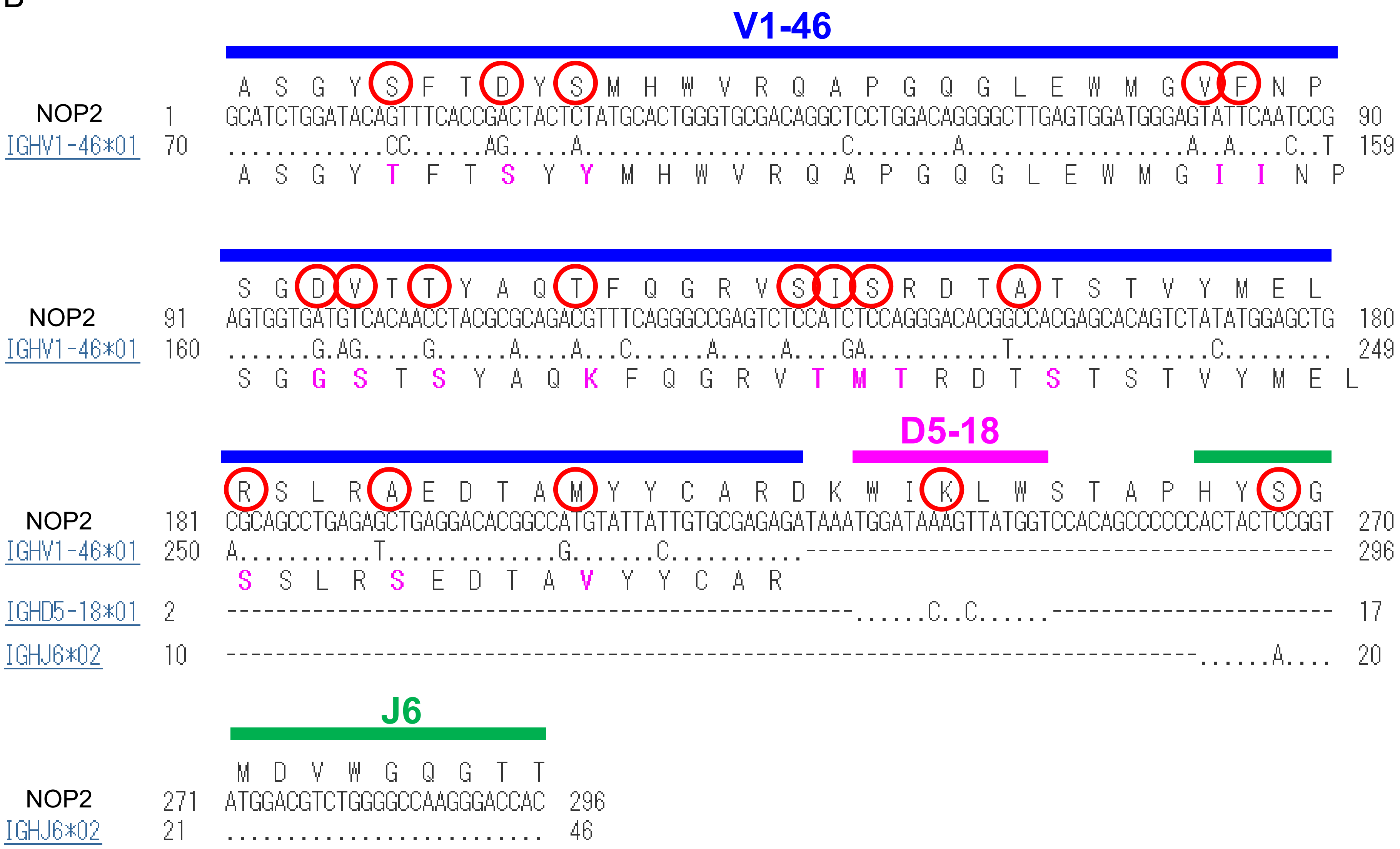
V1-46
A S G Y S F T D Y S M H W V R Q A P G Q G L E W M G V F N P
NOP2 1 GCATCTGGATACAGTTTCACCGACTACTCTATGCACTGGGTGCGACAGGCTCCTGGACAGGGGCTTGAGTGGATGGGAGTATTCAATCCG 90
IGHV1-46*01 70 .............CC......AG.....A....................C........A.................A..A....C..T 159
A S G Y T F T S Y Y M H W V R Q A P G Q G L E W M G I I N P
S G D V T T Y A Q T F Q G R V S I S R D T A T S T V Y M E L
NOP2 91 AGTGGTGATGTCACAACCTACGCGCAGACGTTTCAGGGCCGAGTCTCCATCTCCAGGGACACGGCCACGAGCACAGTCTATATGGAGCTG 180
IGHV1-46*01 160 .......G.AG.....G......A....A...C......A.....A....GA...........T................C......... 249
S G G S T S Y A Q K F Q G R V T M T R D T S T S T V Y M E L
D5-18
R S L R A E D T A M Y Y C A R D K W I K L W S T A P H Y S G
NOP2 181 CGCAGCCTGAGAGCTGAGGACACGGCCATGTATTATTGTGCGAGAGATAAATGGATAAAGTTATGGTCCACAGCCCCCCACTACTCCGGT 270
IGHV1-46*01 250 A...........T..............G.......C...........--- 296
S S L R S E D T A V Y Y C A R
IGHD5-18*01 2 ---......C..C......----------------------- 17
IGHJ6*02 10 --......A.... 20
J6
M D V W G Q G T T
NOP2 271 ATGGACGTCTGGGGCCAAGGGACCAC 296
IGHJ6*02 21 46

Figure S10

C

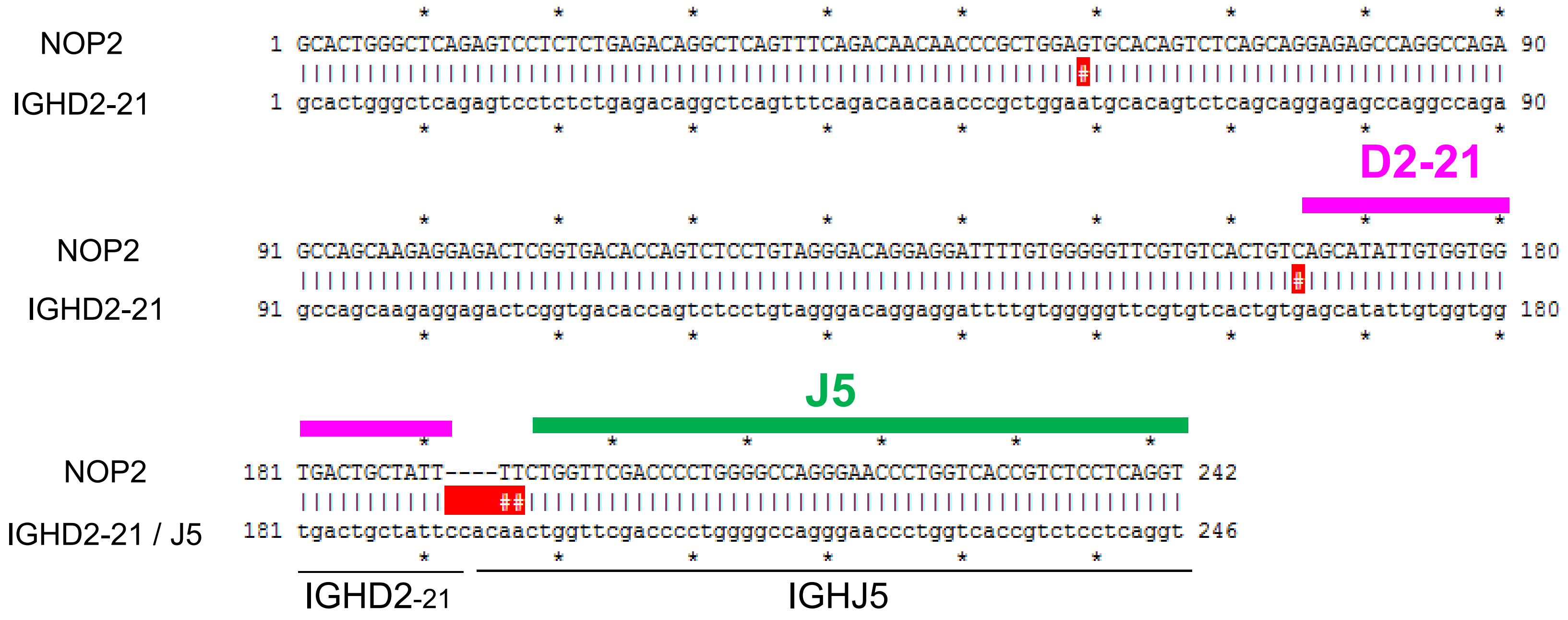
NOP2
1 GCACTGGGCTCAGAGTCCTCTCTGAGACAGGCTCAGTTTCAGACAACAACCCGCTGGAGTGCACAGTCTCAGCAGGAGAGCCAGGCCAGA 90
IGHD2-21
1 gcactgggctcagagtcctctctgagacaggctcagtttcagacaacaacccgctggaatgcacagtctcagcaggagagccaggccaga 90
D2-21
NOP2
91 GCCAGCAAGAGGAGACTCGGTGACACCAGTCTCCTGTAGGGACAGGAGGATTTTGTGGGGGTTCGTGTCACTGTCAGCATATTGTGGTGG 180
IGHD2-21
91 gccagcaagaggagactcggtgacaccagtctcctgtagggacaggaggattttgtgggggttcgtgtcactgtgagcatattgtggtgg 180
J5
NOP2
181 TGACTGCTATT----TTCTGGTTCGACCCCTGGGGCCAGGGAACCCTGGTCACCGTCTCCTCAGGT 242
IGHD2-21 / J5
181 tgactgctattccacaactggttcgacccctggggccagggaaccctggtcaccgtctcctcaggt 246
IGHD2-21
IGHJ5

Figure S10

D KMS-12

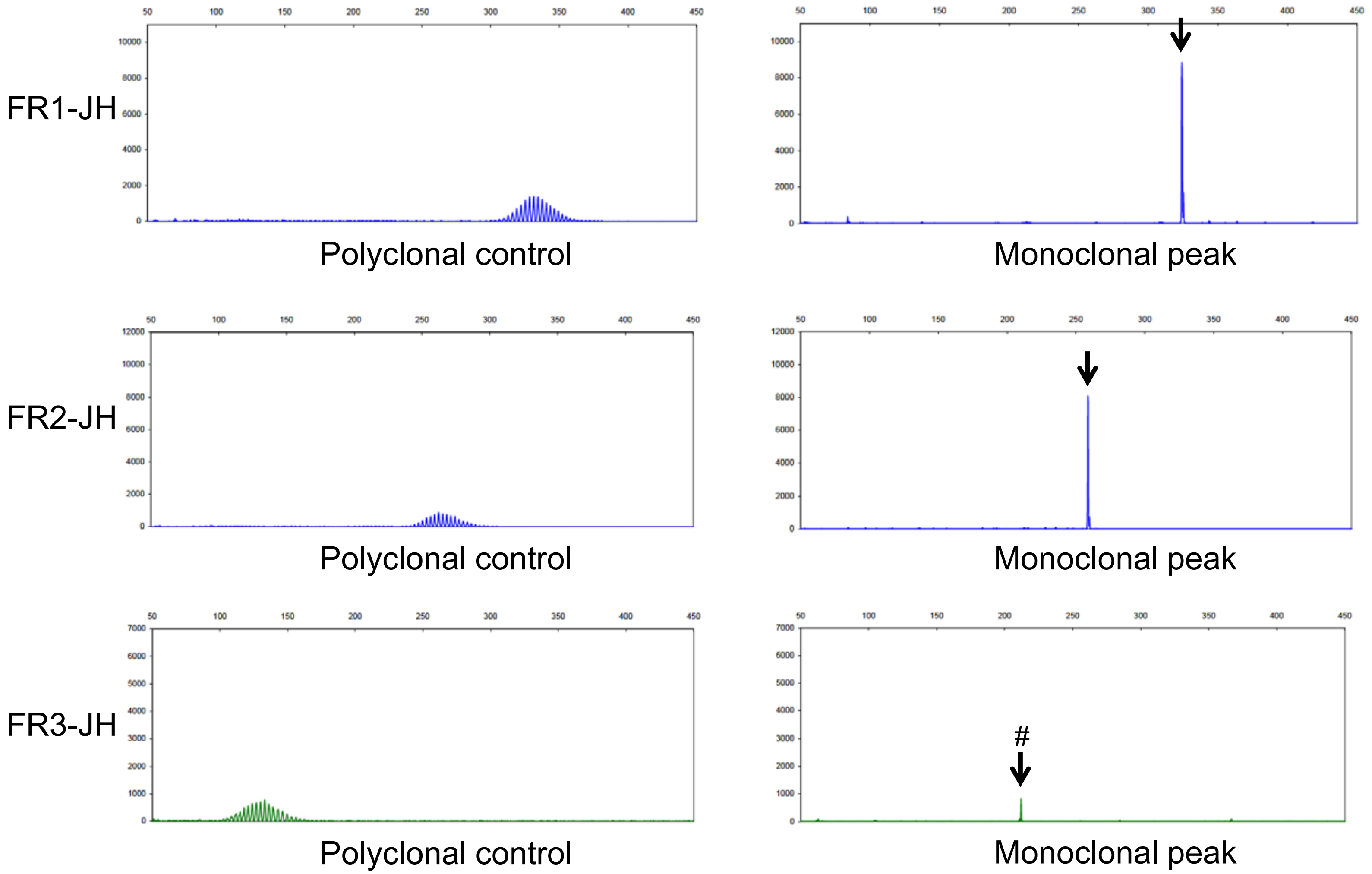

Figure S10

E

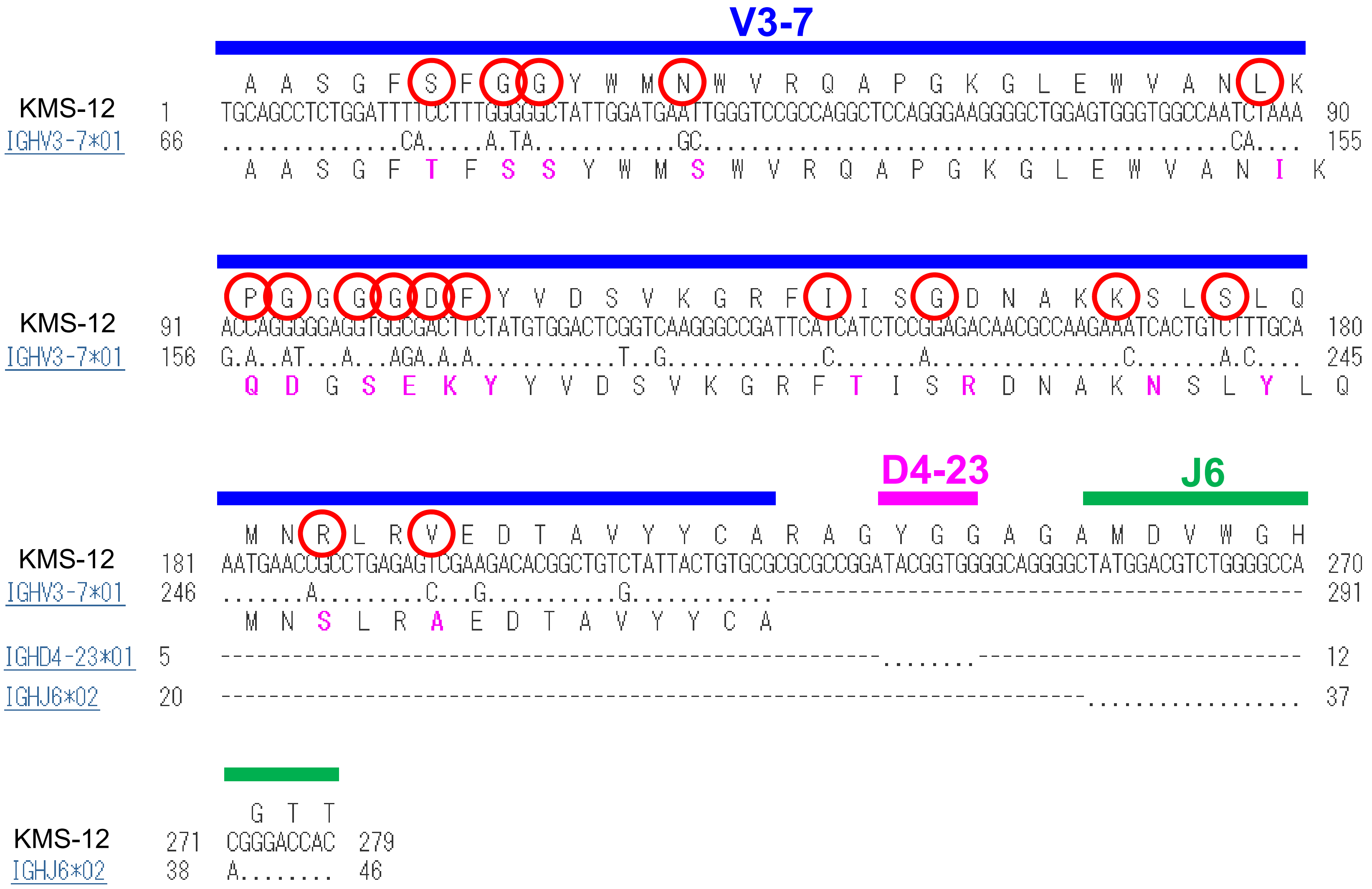
V3-7
A A S G F S F G G Y W M N W V R Q A P G K G L E W V A N L K
KMS-12 1 TGCAGCCTCTGGATTTTTCCTTTGGGGGCTATTGGATGAATTGGGTCCGCCAGGCTCCAGGGAAGGGGCTGGAGTGGGTGGCCAATCTAAA 90
IGHV3-7*01 66 ...............CA.....A.TA............GC..CA.... 155
A A S G F T F S S Y W M S W V R Q A P G K G L E W V A N I K
P G G G G D F Y V D S V K G R F I I S G D N A K K S L S L Q
KMS-12 91 ACCAGGGGGAGGTGGCGACTTCTATGTGGACTCGGTCAAGGGCCGATTCATCATCTCCGGAGACAACGCCAAGAAATCACTGTCTTTGCA 180
IGHV3-7*01 156 G.A..AT...A...AGA.A.A...........T..G.............C.......A...............C.......A.C.... 245
Q D G S E K Y Y V D S V K G R F T I S R D N A K N S L Y L Q
D4-23
J6
M N R L R V E D T A V Y Y C A R A G Y G G A G A M D V W G H
KMS-12 181 AATGAACCGCCTGAGAGTCGAAGACACGGCTGTCTATTACTGTGCGCGCGCCGGATACGGTGGGGCAGGGGCTATGGACGTCTGGGGCCA 270
IGHV3-7*01 246 .......A.........C...G..........G............--- 291
M N S L R A E D T A V Y Y C A
IGHD4-23*01 5 ---........------------------------- 12
IGHJ6*02 20 ---.................. 37
G T T
KMS-12 271 CGGGACCAC 279
IGHJ6*02 38 A........ 46

Figure S10

F

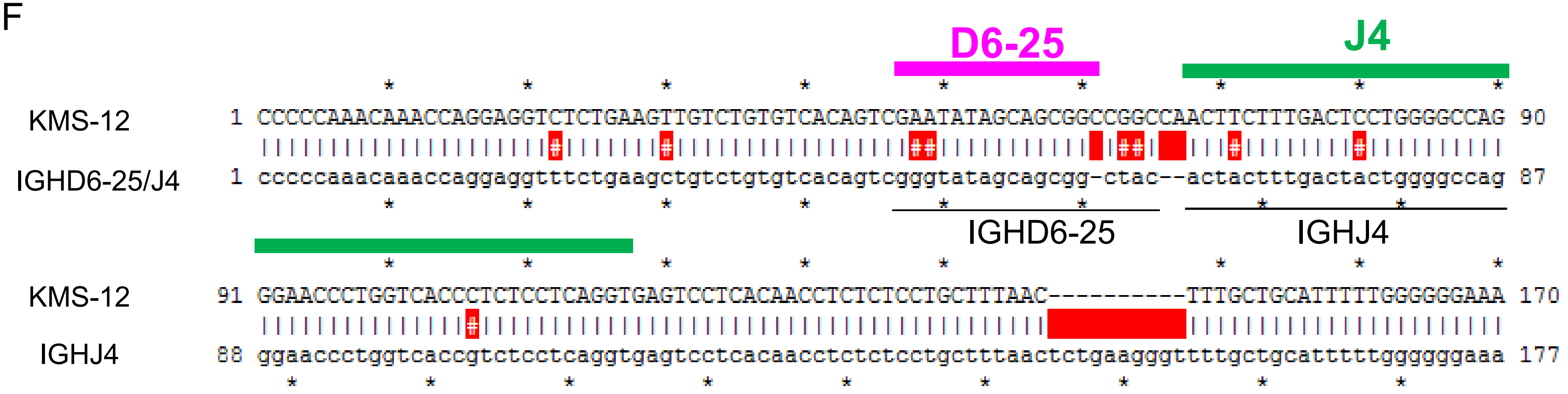
D6-25
J4
KMS-12
1 CCCCCAAACAAACCAGGAGGTCTCTGAAGTTGTCTGTGTCACAGTCGAATATAGCAGCGGCCGGCCAACTTCTTTGACTCCTGGGGCCAG 90
IGHD6-25/J4
1 cccccaaacaaaccaggaggtttctgaagctgtctgtgtcacagtcgggtatagcagcgg-ctac--actactttgactactggggccag 87
IGHD6-25
IGHJ4
KMS-12
91 GGAACCCTGGTCACCCTCTCCTCAGGTGAGTCCTCACAACCTCTCTCCTGCTTTAAC----------TTTGCTGCATTTTTGGGGGGAAA 170
IGHJ4
88 ggaaccctggtcaccgtctcctcaggtgagtcctcacaacctctctcctgctttaactctgaagggttttgctgcatttttgggggggaaa 177
